# Supplementary material for: Optimizing single molecule, real-time sequencing for enhanced characterization of adeno-associated viral vector genomes
Source: Mol Ther Adv. 2026 Apr 1;34(2):201728. doi: 10.1016/j.omta.2026.201728 (PMC13144560; doi:10.1016/j.omta.2026.201728)
Supplement: Document S1. Figures S1–S5 and Tables S1 and S2 [file mmc1.pdf]

OMTA, Volume *34*

Supplemental information

Optimizing single molecule, real-time sequencing for enhanced characterization of adeno-associated viral vector genomes

Julia Manz, Raphael Ruppert, Markus Haindl, Jürgen Hubbuch, and Johannes Pschirer

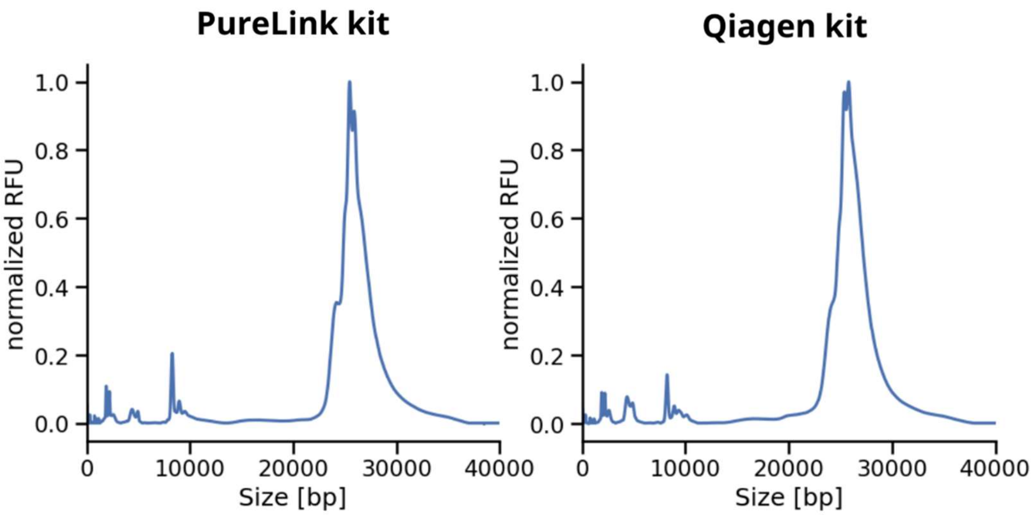

Figure S1: Comparison between the PureLink^{TM} Viral RNA/DNA Mini Kit and the QIAamp MinElute Virus Spin Kit from Qiagen

rAAV2-mGL DNA was extracted using either the PureLink^{TM} Viral RNA/DNA Mini Kit or the QIAamp MinElute Virus Spin Kit. To compare the performance of both kits, the obtained ssDNA was analyzed on a Fragment Analyzer 5300. The RFU values were normalized to the peak with the highest intensity.

A

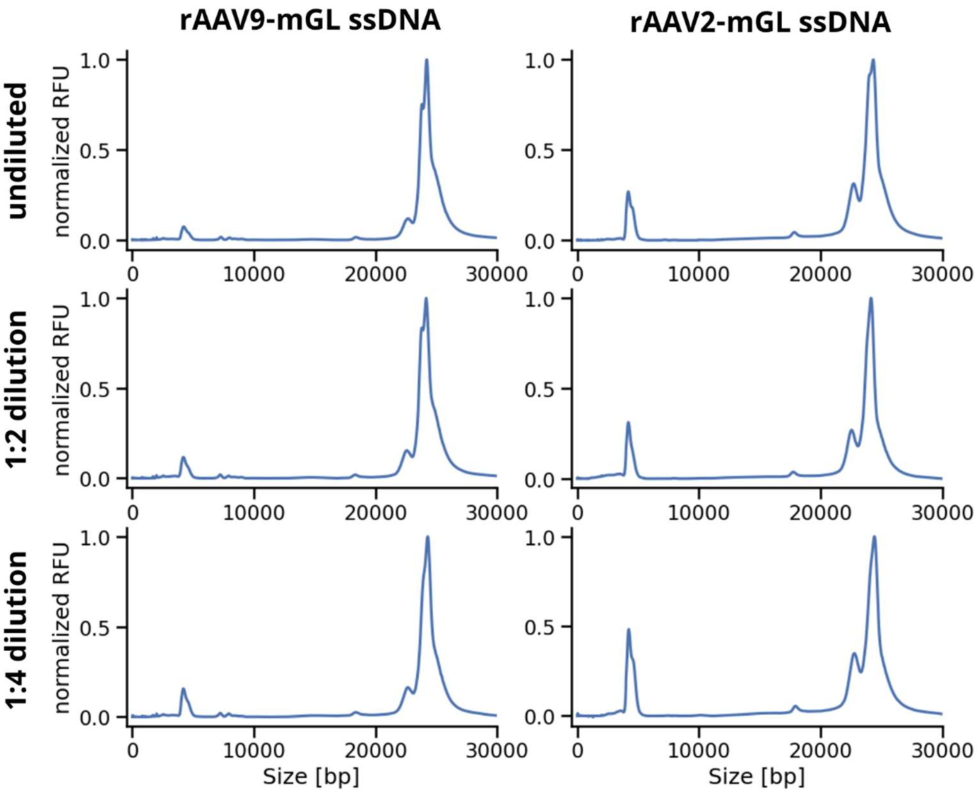
rAAV9-mGL ssDNA
rAAV2-mGL ssDNA
undiluted
1:2 dilution
1:4 dilution
normalized RFU
1.0
0.5
0.0
0
10000
20000
30000
Size [bp]

B

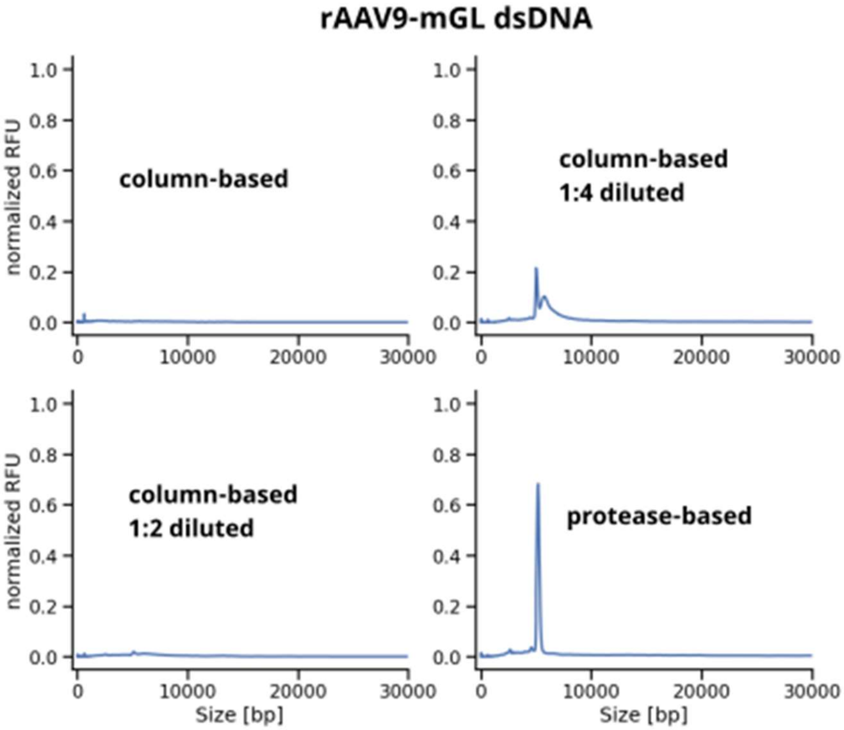
rAAV9-mGL dsDNA
column-based
column-based
1:4 diluted
column-based
1:2 diluted
protease-based
normalized RFU
1.0
0.8
0.6
0.4
0.2
0.0
0
10000
20000
30000
Size [bp]

Figure S2: Investigation of the influence of the titer on the column-based ssDNA extraction

(A) ssDNA from undiluted, 1:2, and 1:4 diluted rAAV9-mGL and rAAV2-mGL samples was extracted using the column-based QIAamp MinElute Virus Spin Kit and analyzed on a Fragment Analyzer 5300. The expected length is approximately 4.5 kb for both vectors. The RFU values were normalized to the peak with the highest intensity. (B) rAAV9-mGL ssDNA was extracted via either protease treatment or the column-based approach. For the latter, different dilutions of the vector were used. The resulting ssDNA served as template for dsDNA synthesis using Bst 3.0. The obtained dsDNA was subsequently analyzed on a Fragment Analyzer 5300.

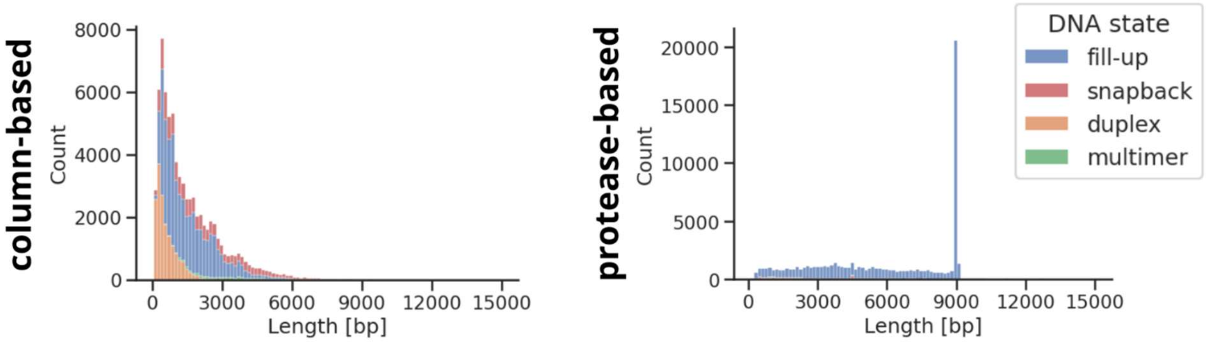

Figure S3: Read length distributions obtained from rAAV2-mGL ssDNA extracted via the column-based or protease-based protocol

rAAV2-mGL ssDNA was extracted using either the column-based QIAamp MinElute Virus Spin Kit or a protease treatment. The plot shows the distribution of the read lengths mapping to the reference plasmid. The DNA states are depicted in different colors. Due to the method used for dsDNA generation, full-length reads are expected to appear as fill-ups with a length of approximately twice the rAAV genome (~9.1 kb).

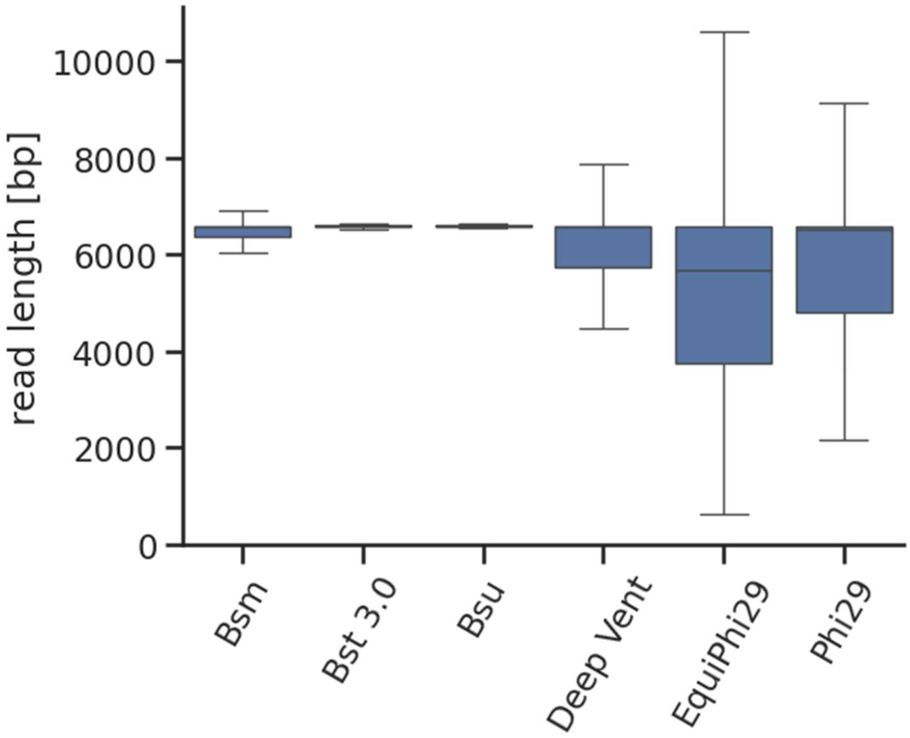

Figure S4: Read length distributions of rAAV2-GFP ssDNA filled up with different polymerases

rAAV2-GFP ssDNA was converted to dsDNA using various polymerases. After library preparation and sequencing, the obtained reads mapping primarily to the reference plasmid were analyzed with regard to their length distribution. The expected read length is ~6.7 kb.

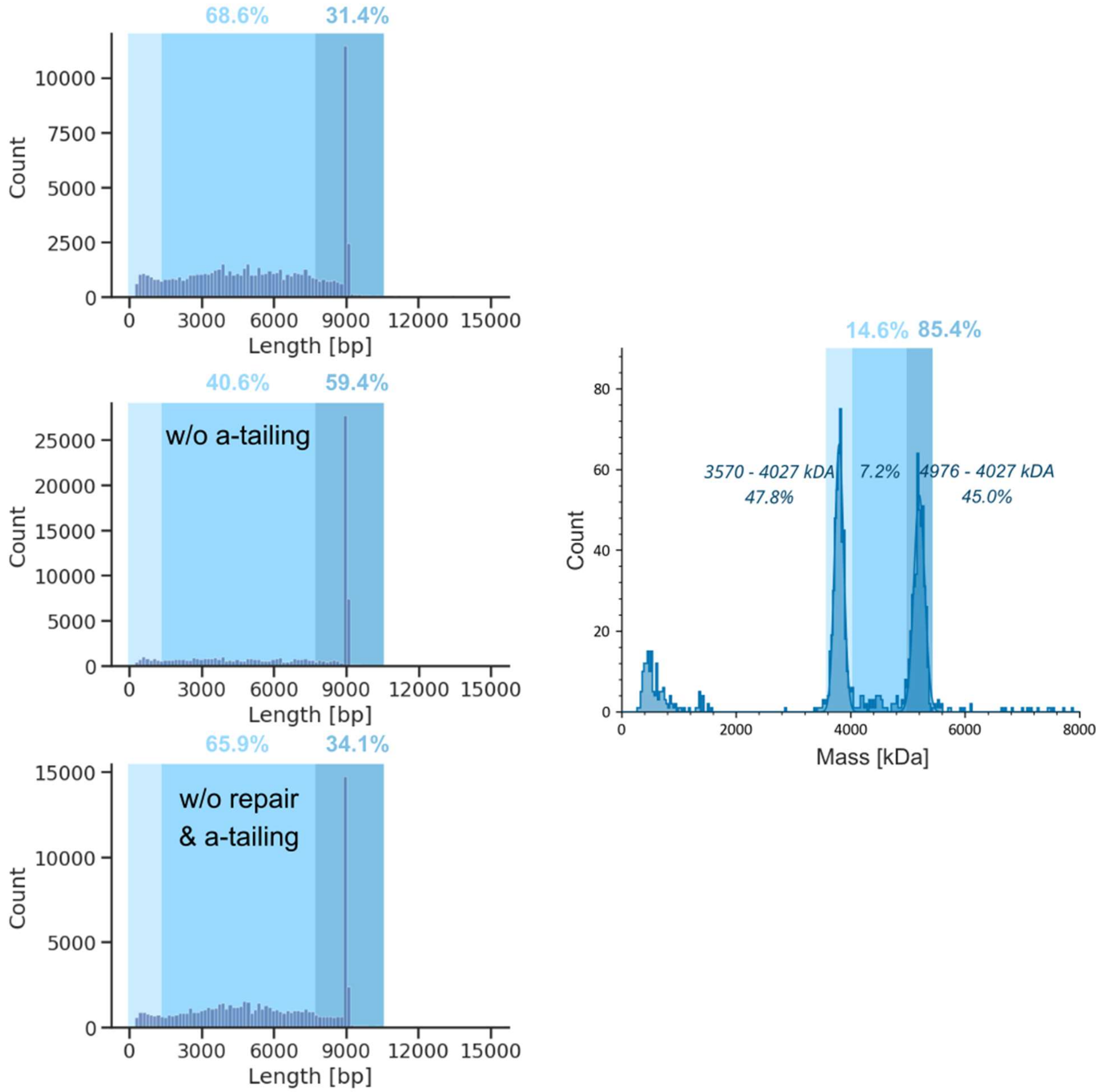

Figure S5: Comparison of NGS data with mass photometry data

rAAV9-mGL ssDNA extracted via protease treatment was prepared for sequencing according to PacBio's protocol, omitting the 65°C step for A-tailing or by skipping the entire repair and A-tailing procedure. After SMRT sequencing, read length distributions were analyzed (left). Additionally, mass photometry was used to quantify the percentage of empty (light blue), partial (mid blue) and full capsids (dark blue) (right). The boundaries obtained via mass photometry were transferred to the NGS data. The percentages of the partial and full fractions excluding the empty one are depicted above each diagram.

Table S1: Percentage of bases fitting to different reference sequences and percentage of full-length rAAV genomes in rAAV2-mGL samples with and without DNase treatment prior to genome extraction

rAAV: recombinant adeno-associated viral vector; pRepCapHelper: generic sequence of contamination with repcap or helper plasmids

		DNase treatment	no DNase treatment
% bases	rAAV	97.5	97.6
	plasmid backbone	1.7	1.6
	pRepCapHepler	0.7	0.7
	human	0.1	0.1
	E. coli	0.0	0.0
	unmapped	0.0	0.0
% full length rAAV genomes		30.0	29.7

Table S2: Percentage of bases fitting to different contamination-related reference sequences

rAAV: recombinant adeno-associated viral vector; pRepCapHelper: generic sequence of contamination with repcap or helper plasmids

rAAV	Repair & A-Tailing	Contamination	% bases
rAAV2-GFP	yes	plasmid backbone	3.1
		pRepCapHelper	0.2
		human	0.2
		E. coli	0.1
		unmapped	0.0

	only repair step	plasmid backbone	1.8
		pRepCapHelper	0.1
		human	0.1
		E. coli	0.1
		unmapped	0.0
	no	plasmid backbone	2.5
		pRepCapHelper	0.2
		human	0.1
		E. coli	0.1
		unmapped	0.0
rAAV9-mGL	yes	plasmid backbone	0.2
		pRepCapHelper	4.6
		human	0.3
		E. coli	0.0
		unmapped	0.0
	only repair step	plasmid backbone	0.2
		pRepCapHelper	3.1
		human	0.1
		E. coli	0.0
		unmapped	0.0
	no	plasmid backbone	0.2
		pRepCapHelper	4.6
		human	0.2
		E. coli	0.0
		unmapped	0.0
