# Supplementary material for: Optimizing single molecule, real-time sequencing for enhanced characterization of adeno-associated viral vector genomes
Source: Mol Ther Adv. 2026 Apr 1;34(2):201728. doi: 10.1016/j.omta.2026.201728 (PMC13144560; doi:10.1016/j.omta.2026.201728)
Supplement: Document S2. Article plus supplemental information [file mmc2.pdf]

Molecular Therapy
Advances
Original Article

Optimizing single molecule, real-time sequencing for enhanced characterization of adeno-associated viral vector genomes

Julia Manz,[1] Raphael Ruppert,[1] Markus Haindl,[1] Jürgen Hubbuch,[2] and Johannes Pschirer[1]

[1]Gene Therapy Technical Development, Roche Diagnostics GmbH, Nonnenwald 2, Penzberg 82377, Germany; [2]Institute of Process Engineering in Life Sciences, Section IV: Biomolecular Separation Engineering, Karlsruhe Institute of Technology, Karlsruhe 76131, Germany

Monitoring the payload of recombinant adeno-associated viral vectors (rAAVs) used for gene therapy applications is essential. Long-read sequencing is a promising method to confirm not only the identity of the packaged DNA but also to assess its integrity with regard to truncations, deletions, insertions, and chimeras. Given its high accuracy, even in regions that are notoriously difficult to sequence, such as the rAAV's inverted-terminal repeats, single molecule, real-time (SMRT) sequencing by Pacific Biosciences might be well suited for quality control purposes. However, the library preparation workflow involves multiple steps that could potentially introduce biases and thereby misrepresent the sample composition. Here, we present a systematic study investigating the influence of several steps on the quality of the output data. Based on these insights, we introduce an adapted protocol to enhance the SMRT technology for accurate and reproducible in-depth characterization of the ssDNA payload in rAAVs.

INTRODUCTION

Recombinant adeno-associated viral vectors (rAAVs) offer several advantages, such as low pathogenicity and long-term episomal transgene expression, making them promising delivery tools for gene therapy.[1,2] To date, they have already been explored in numerous clinical trials and approved in several drugs.[1,3] While rAAV-based gene therapies have improved patients' lives, fatal outcomes have also occurred.[4,5] Consequently, rigorous and improved quality control (QC) is essential. For rAAVs, this involves characterizing both the capsid and the single-stranded DNA (ssDNA) payload. Ideally, the capsid contains either a plus or minus strand of the full-length expression cassette, flanked by inverted-terminal repeats (ITRs).[6] However, truncated, mutated, or rearranged genomes may also be present. In addition, the packaging of contaminating DNA from other sources, such as the producer cells or plasmids, has been reported.[7] Sanger sequencing is the current gold standard for identity testing, whereas quantitative polymerase chain reaction (qPCR) or digital PCR (dPCR) are used for the detection of predefined contaminations.[8,9] Due to its unique features, next-generation sequencing (NGS) has the potential to combine both, identity testing and "unbiased" identification of contaminants in a single assay.[10] Short-read sequencing platforms, such as the Illumina technology, can produce reads with a length of up to only 300 base pairs (bp),[11] making the detection of concatemers, chimeras, and truncations difficult. Long-read sequencing can overcome this hurdle by achieving read lengths of several kilobases (kb), thereby covering the entire rAAV genome sequence (~4.7 kb)[12] in a single read. Due to its high accuracy,[13,14] single molecule, real-time (SMRT) sequencing by Pacific Biosciences (PacBio) seems to be a promising technology. During library preparation, hairpin adapters are ligated to both ends of a double-stranded DNA (dsDNA) template, resulting in a circular molecule called SMRTbell template. Next, a sequencing primer and a polymerase are bound to the hairpin, and the complex is loaded onto an SMRT cell. During the sequencing run, the polymerase extends the primer by incorporating fluorescently labeled deoxynucleotide triphosphates (dNTPs). Since the SMRTbell template is circular, the polymerase synthesizes a very long DNA molecule that contains both strands multiple times (called "passes").[15,16] With 10%–15%, the error rate of the sequencing polymerase is quite high.[13] However, the errors are randomly distributed and by generating a consensus sequence from the long polymerase read, a high accuracy can be achieved.[17] The final output contains only high fidelity (HiFi) reads with a minimum accuracy of 99% and a median accuracy of 99.9%.[18]

Although the high accuracy of this platform is favorable for mutation detection, the library preparation involves multiple different steps that can introduce biases and have an effect on the integrity of the rAAV ssDNA. The standard protocol is as follows: first, to obtain the required dsDNA for sequencing, the vector genomes must be extracted from the capsids first. Next, the ssDNA has to be converted to dsDNA which is achieved by thermal annealing of plus and minus genomes, according to PacBio's protocol. In the repair and A-tailing step, damages such as abasic sites, nicks, and modified bases are repaired, the DNA ends are blunted, and a single A-overhang is added so that in the next step barcoded hairpin adapters with

Received 27 October 2025; accepted 27 March 2026;
https://doi.org/10.1016/j.omta.2026.201728.

Correspondence: Johannes Pschirer, Gene Therapy Technical Development, Roche Diagnostics GmbH, Nonnenwald 2, Penzberg, Bavaria 82377, Germany.
E-mail: johannes.pschirer@roche.com

Published by Elsevier Inc. on behalf of The American Society of Gene and Cell Therapy.
This is an open access article under the CC BY license (http://creativecommons.org/licenses/by/4.0/).

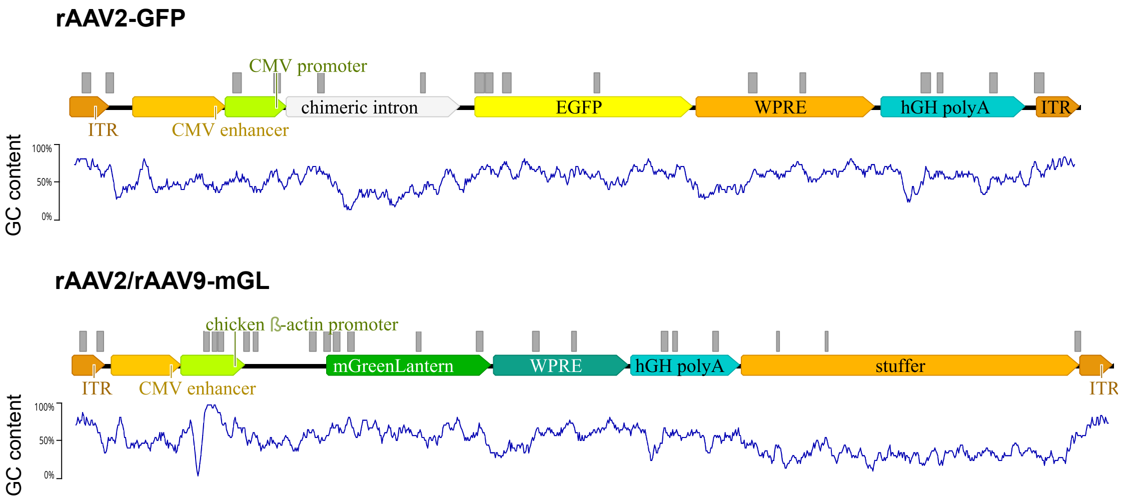

Figure 1. Schematic representation of the vector genomes used

Annotated vector genomes of rAAV2-GFP (top) and rAAV2/rAAV9-mGL (bottom) are shown. Putative quadruplex forming G-rich sequences are indicated by gray boxes above the respective genomes, with the GC content depicted in the following text.

single T-overhangs can be ligated to produce circular molecules.[19] Subsequently, a nuclease treatment is performed to remove "imperfect", i.e., non-circular SMRTbell templates and remaining adapters. After sample pooling, sequencing primers and polymerases are bound to the library and the complexes are loaded onto the sequencer. Between the individual steps, several bead cleanups are performed to get rid of the enzymes and adjust the volume and buffer for the subsequent reaction.[20] Following this standard protocol is suitable for identity testing. However, in terms of QC, the library preparation procedure needs further improvements in order to reduce biases and effects on the input rAAV ssDNA. Nevertheless, there are almost no publications dealing with this issue.[21] To close this gap and to improve AAV sequencing, we demonstrate the impact of individual steps and propose an adapted library preparation protocol.

RESULTS

rAAV DNA extraction

To investigate the influence of various steps during rAAV library preparation and to improve the protocol, two different vectors were used. The first one, referred to as rAAV2-GFP, comprises an AAV2 capsid containing a payload of 3,342 bases encoding the enhanced green fluorescent protein (EGFP) and has a titer of $\sim 6 \times 10^{12}$ vector genomes (vg)/mL. The second, rAAV9-mGL, contains a genome of 4,565 bases encoding mGreenLantern (mGL) inside an AAV9 capsid and has a titer of $\sim 2 \times 10^{13}$ vg/mL. For verification purposes, a third rAAV, rAAV2-mGL ($\sim 2 \times 10^{13}$ vg/mL), identical to rAAV9-mGL, but with an AAV2-derived capsid, was utilized. A comprehensive overview of the vector genomes used is provided in Figure 1. Analysis of the two different payloads using the QGRS mapper tool[22] revealed 16 putative quadruplex-forming sequences without overlaps (gray boxes in Figure 1) and 743 including overlaps for rAAV2-GFP. By contrast, the rAAV2/rAAV9-mGL genome showed 22 hits without overlaps and 8,269 hits including overlaps and these exhibited higher G-scores than those of rAAV2-GFP.

The initial step in PacBio's rAAV sequencing workflow is DNase I treatment without heat inactivation to prevent premature genome release. However, as the vectors utilized were extensively purified during production (see rAAV constructs) and since comparable levels of contamination were observed irrespective of whether the digest was performed prior to sequencing or omitted (Table S1), we decided to skip this step for the subsequent experiments presented here. Nevertheless, DNase I treatment might be necessary for rAAV samples of lower purity. As shown in Table S1, we did not observe any influence of the DNase digest on the integrity of the encapsidated DNA (30% full-length genomes in both cases). Since our experiment was conducted with an AAV2-derived capsid, which is the least thermostable,[23] other serotypes should also be compatible with the DNase I treatment as suggested by PacBio.

For the next step, vector genome extraction, the protocol suggests using the column-based PureLink Viral RNA/DNA Mini Kit from Thermo Fisher Scientific if the loss of fragments <200 bp is not a concern; otherwise, a pronase treatment followed by phenol/chloroform extraction, as described by Tran et al.,[24] is recommended. In a preliminary experiment, we compared the PureLink Viral RNA/DNA Mini Kit with the QIAamp MinElute Virus Spin Kit from QIAGEN which is also column based and did not observe any substantial differences (Figure S1). For the experiments presented here, the Qiagen kit was employed (hereafter referred to as column-based protocol). The second option, the extraction protocol by Tran et al., involves a pronase digest for 4 h followed by phenol/chloroform extraction, which is time-consuming and can be hazardous.[24,25] Therefore, we sought an alternative. Since the Qiagen protocol also

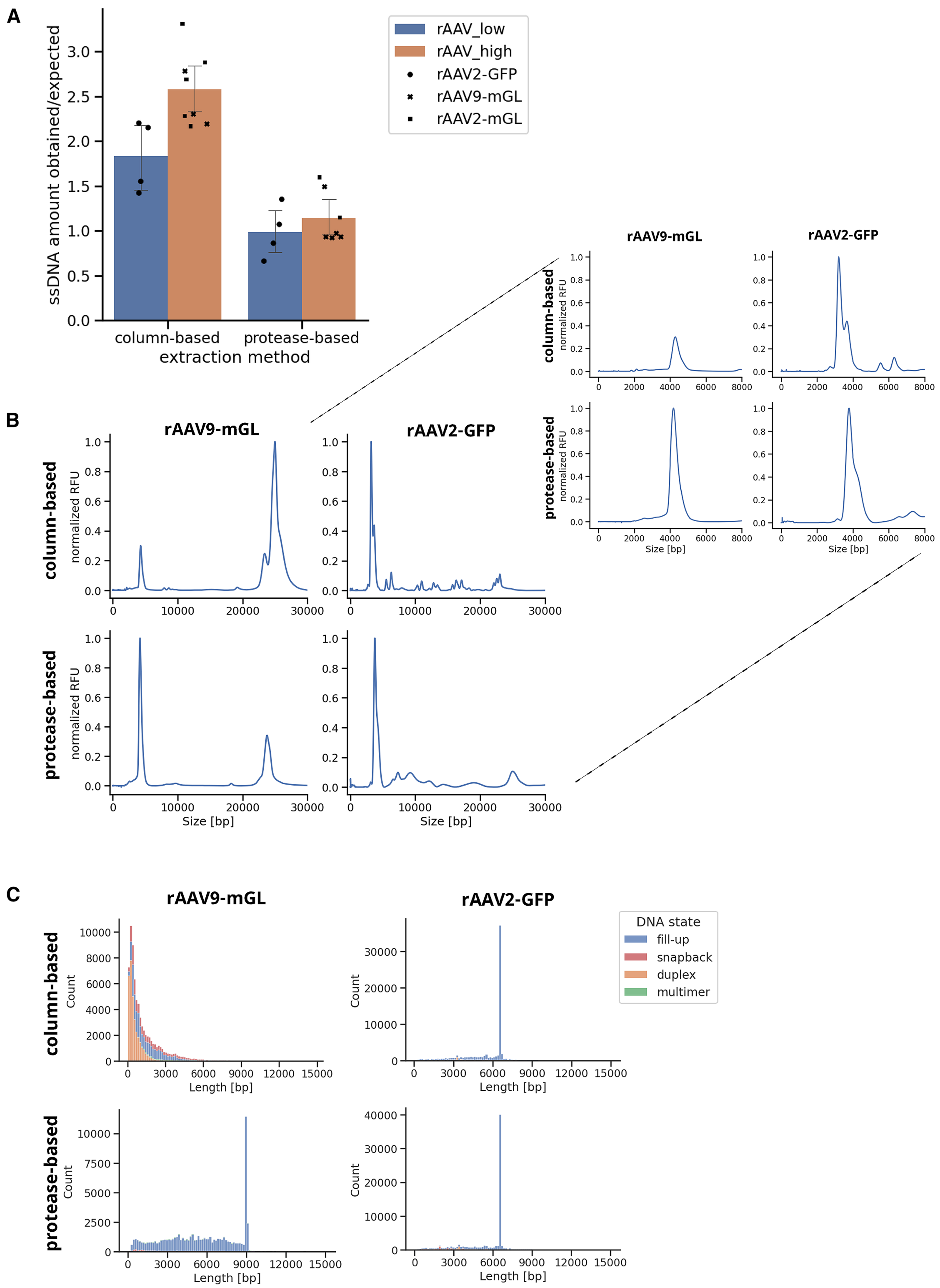

Figure 2. Comparison of column- and protease-based rAAV ssDNA extraction

ssDNA from rAAV2-GFP, rAAV9-mGL, and rAAV2-mGL was extracted using either the column-based QIAamp MinElute Virus Spin Kit or a protease treatment. (A) ssDNA amount from multiple extractions was measured with the Qubit ssDNA assay kit, and the ratio to the expected DNA amount was calculated. rAAV_low refers to rAAV2-GFP

(legend continued on next page)

involves a protease treatment for 15 min prior to column loading, we reasoned that we could perform only the digest, substituting the kit's buffer with isothermal amplification buffer II—the buffer needed in the next step (see double-strand synthesis)—or even perform the digest without the addition of any buffer (referred to as protease protocol).

Following extraction, the ssDNA concentration was measured using the Qubit ssDNA assay kit and compared to the maximum possible yield calculated based on the respective titer. Although the measured concentration was about 1.8 times the expected amount for "low" concentrated rAAV2-GFP samples ($\sim 6 \times 10^{12}$ vg/mL) using the column-based approach, it exceeded 2.5 times the expected amount for "high" concentrated rAAV9-mGL vectors ($\sim 2 \times 10^{13}$ vg/mL). To exclude any effect attributable to the capsid type, rAAV2-mGL was used for verification. Since both vectors have the same vg titer and the measured ssDNA concentrations were similar, they were consolidated as AAV_high in Figure 2A. In contrast, when the protease protocol was used, the ratio of the obtained to expected ssDNA amount was about one, regardless of the titer or the use of buffer (rAAV2-GFP with buffer: 0.9, without buffer: 1.0; rAAV9-mGL with buffer: 0.9, without buffer: 0.9). Again, the results of rAAV9-mGL were verified with the rAAV2-mGL (Figure 2A). To investigate the influence of different DNA states (i.e., ssDNA and dsDNA) on the output of the Qubit ssDNA assay kit, a spiking experiment was performed. To this end, different amounts of the Qubit dsDNA and ssDNA standards were mixed and measured with the ssDNA assay kit (Table 1). The obtained values corresponded to the input amount of ssDNA plus 4.5 times the amount of spiked-in dsDNA.

To further analyze the extracted rAAV ssDNA, it was run on a Fragment Analyzer 5300 (Figure 2B). The main peak was at the expected length of approximately 3.3 kb for rAAV2-GFP, irrespective of the extraction method used. The detected difference between the two extraction methods might be attributed to the sizing accuracy of the Fragment Analyzer and differences in unresolved secondary structures, e.g., in the ITRs. By contrast, the rAAV9-mGL sample extracted with the column-based kit showed only a minor peak at the expected length of 4.5 kb and an additional, highly prominent one at about 25 kb, which is most likely caused by multimers. Using protease for the ssDNA extraction increased the intensity of the monomeric peak while decreasing the signal of the multimers. To investigate whether this observation was due to the different vector genome titers of rAAV2-GFP and rAAV9-mGL, 1:2 and 1:4 dilutions of rAAV9-mGL were extracted with the Qiagen kit and loaded onto the Fragment Analyzer. With increasing dilution, the ratio between monomeric and multimeric signal slightly increased but was not anyway near to that of rAAV2-GFP. The same trend as for rAAV9-mGL could be observed for rAAV2-mGL (Figure S2A). In line with these results, conversion into dsDNA using the 3′ ITR as a primer failed to yield a product for the undiluted or 1:2 diluted samples but succeeded with the 1:4 dilution. Double-strand synthesis was most efficient for the sample extracted via protease treatment (Figure S2B).

Moreover, the obtained ssDNA was used as input for library preparation, which includes several bead cleanup steps. The magnetic beads used are suspended in a buffer containing salts and polyethylene glycol (PEG). If the concentration of these two components—determined by the used volume of the solution—is high enough, DNA fragments of all lengths can bind non-specifically to the cleanup beads. However, larger DNA molecules display a higher affinity. Therefore, with lower concentrations of PEG and salts, only larger nucleic acids with high affinity can bind to the beads.[26] Consequently, to minimize DNA loss, we increased the bead ratio during the cleanups from 1.3× volume/volume (v/v) to 2× v/v. The output data of the sequencing run were analyzed with respect to read lengths and DNA state. Duplexes result from annealed DNA strands, whereas fragments containing ITRs that can serve as primers—filled up resulting in molecules with an ITR in the symmetry axis—are defined as fill-ups. Snapbacks[27] are similar to fill-ups but lack an ITR in the symmetry axis. Finally, reads having multiple supplementary alignments, i.e., being split into several parts because they fit non-adjacent positions in the references, are classified as multimers. These could include, for instance, chimeric genomes, unresolved multimeric genomes from rolling-hairpin replication, or artifacts of the second-strand synthesis. Strikingly, although the sequencing results were very similar for rAAV2-GFP DNA extracted with either the kit or the protease, there was a substantial difference between the extraction methods for rAAV9-mGL. While the column-based approach resulted in a high proportion of duplex DNA and no full-length reads, the protease digest resulted predominantly in fill-ups and about 18% full-length genomes (Figure 2C). These findings were reproducible with rAAV2-mGL (Figure S3; Table 2). Consequently, extracting the rAAV ssDNA via protease digest appears superior.

Conversion of ssDNA to dsDNA

After extraction, the standard PacBio protocol suggests converting the obtained ssDNA to dsDNA via thermal annealing of plus and minus genomes.[20] However, this approach may introduce biases and lead to a loss of information. On the one hand, truncated

with a titer of $\sim 6 \times 10^{12}$ vg/mL and rAAV_high comprises the results of rAAV9-mGL and rAAV2-mGL with a titer of $\sim 2 \times 10^{13}$ vg/mL. Shown are the average ratios with error bars representing 95% confidence intervals. $n = 4$ for rAAV_low column and protease based, $n = 5$ for rAAV_high column based, $n = 6$ for rAAV_high protease based. (B) The extracted ssDNA was analyzed using a Fragment Analyzer 5300. One representative electropherogram is shown per condition. The expected lengths are approximately 3.3 kb for rAAV2-GFP and 4.5 kb for rAAV9-mGL. A magnified view of the size distributions up to 8 kb is provided on the right. RFU values were normalized to the peak with the highest intensity. (C) Distribution of the read lengths mapping to the reference plasmids. The DNA states are depicted in different colors. Due to the used method for dsDNA generation, full-length reads should be fill-ups with a length of approximately twice that of the rAAV genome, which would be ~9.1 kb for rAAV9-mGL and ~6.7 kb for rAAV2-GFP. RFU, relative fluorescence unit.

Table 1. Evaluation of the influence of dsDNA on the output of the Qubit ssDNA assay kit

Used dsDNA amount [ng/μl]	10	6.67	5	3.33	0
Used ssDNA amount [ng/μl]	0	**6.67**	10	13.33	20
Measurement 1 [ng/μl]	46.8	33.6	31.0	26.2	20.0
Measurement 2 [ng/μl]	48.6	33.0	31.4	27.6	23.0
Average [ng/μl]	47.7	33.3	31.2	26.9	21.5

dsDNA, double-stranded DNA; ssDNA, single-stranded DNA.

Table 2. Percentages of full-length rAAV reads obtained from different rAAVs, ssDNA extraction methods, and the inclusion or exclusion of the repair and A-tailing step during library preparation

rAAV	Extraction method	Repair & A-tailing step	% full-length
rAAV2-GFP	column based	yes	37
rAAV2-GFP	column based	only repair step	51
rAAV2-GFP	column based	no	41
rAAV2-GFP	protease	yes	40
rAAV2-GFP	protease	only repair step	51
rAAV2-GFP	protease	no	43
rAAV2-mGL	column based	yes	0
rAAV2-mGL	protease	yes	18
rAAV9-mGL	Ccolumn based	yes	0
rAAV9-mGL	column based	only repair step	0
rAAV9-mGL	column based	no	0
rAAV9-mGL	protease	yes	18
rAAV9-mGL	protease	only repair step	47
rAAV9-mGL	protease	no	22

rAAV, recombinant adeno-associated viral vector; ssDNA, single-stranded DNA.

genomes might not find an annealing partner and therefore be digested during the nuclease treatment at the end of library preparation. On the other hand, they could anneal with another molecule of not the exact same size. During the repair step, blunt ends are created by filling in 5′ overhangs and removing 3′ overhangs. Hence, if the two annealed molecules are not identical in size, this leads to misrepresentation of partial genomes in the data. In contrast, if the size differs considerably between the annealing partners, the repair enzymes might be unable to generate blunt ends. In this case, no adapters could be ligated, and thus, again, the molecules would be digested by the nuclease. Therefore, we opted to replace thermal annealing with double-strand synthesis using the ITR with the free 3′–OH group as a primer. In theory, any encapsidated DNA should contain at least an ITR at the 3′ end, since ITRs are essential for the packaging of DNA into capsids,[6] and the small Rep proteins are reported to channel the ssDNA in 3′→5′ direction through the 5-fold pore into the capsid.[28–30] For dsDNA synthesis, a polymerase with strand displacement function but lacking 5′→3′ exonuclease activity is required, as it must displace the 5′ ITR without digesting it. As part of a previous study, we evaluated several such polymerases including Bst 3.0, Bsm, Bsu, Deep Vent, EquiPhi29, and phi29 (Figure S4). Bst 3.0 and Bsu were superior in converting rAAV ssDNA to dsDNA resulting in almost no artifactual species, e.g., fragments substantially exceeding the rAAV packaging capacity. Since the obtained dsDNA amount was higher and closer to the expected value using Bst 3.0, we decided to proceed with this polymerase. With the 3′ ITR serving as a primer, the resulting molecules have only one open end meaning only one hairpin adapter can be ligated during library preparation (Figure 3A). As an SMRT read is defined as the sequence between two adapters, our SMRTbell templates are expected to result in twice the read length of the original molecules (see, for example, Figure 2C).

In order to inactivate the protease and to resolve annealed genomes prior to double-strand synthesis, the DNA samples were heated to 95°C and immediately chilled on ice. Interestingly, adding the polymerase buffer and $MgSO_4$ before or after this heat shock had a substantial impact on the DNA state as detected by NGS (Figure 3B). While adding these components right from the beginning to rAAV2-GFP ssDNA resulted in a high proportion of duplexes, omitting them during the heat shock yielded almost 100% fill-ups, irrespective of the extraction method. Representative read length plots including the DNA state are shown for the Qiagen Kit in Figure 3B. Concerning rAAV9-mGL and rAAV2-mGL, no conclusion could be drawn for column-based extraction, as this approach did not yield any full-length reads (Figure 2C top left and Figure S3 left). With the protease treatment, no duplex DNA was detected, irrespective of whether buffer and $MgSO_4$ were added before or after the heat shock. To further investigate this, the effect of these components on the melting temperature was analyzed. Indeed, the melting temperature of a 6.2 kb linearized plasmid, measured on a LightCycler using SYBR Green I, increased from 91°C to 94.5°C upon addition of the buffer and $MgSO_4$. Based on these results, we would recommend extracting the rAAV ssDNA via protease digestion in the absence of buffer and adding all components for the dsDNA synthesis only after the heat shock.

Repair and A-tailing

Following conversion to dsDNA and a bead cleanup, the samples undergo a repair and A-tailing step. Abasic sites, nicks, and base modifications are repaired, the DNA ends are blunted, and a single A-overhang is introduced.[19] Since our rAAV library preparation workflow starts with ssDNA and utilizes a polymerase for second-strand synthesis, the occurrence of nicks is unlikely, as they would terminate the synthesis process. Furthermore, Bst 3.0 naturally incorporates an A at the 3′ end. Therefore, we questioned whether this step is necessary and if it might introduce some kind of bias. PacBio's protocol involves incubation at 37°C for 30 min for DNA repair, followed by 65°C for 5 min for A-tailing. We compared the standard protocol with omitting the 65°C step, and thus the A-tailing, and with skipping the entire

A

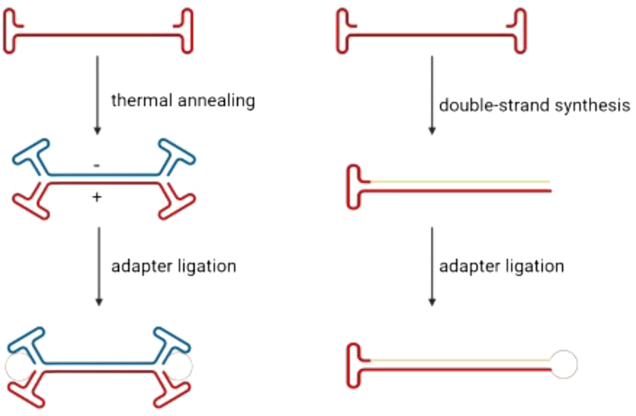

B

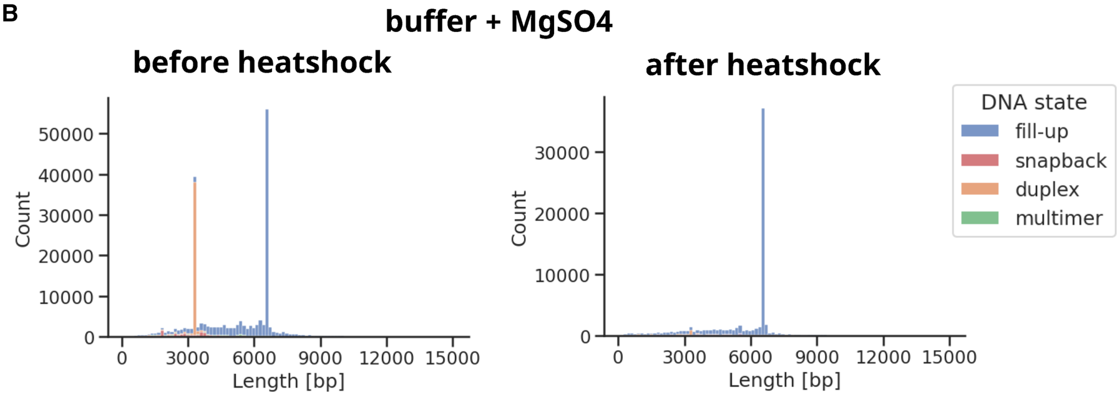

Figure 3. Schematic of rAAV SMRTbell templates and influence of the time point of adding buffer and $MgSO_4$ on the DNA state of the library molecules
(A) Following thermal annealing of rAAV ssDNA, hairpin adapters can be ligated to both ends of the resulting dsDNA. In contrast, double-strand synthesis using the 3′ ITR as a primer results in only one open end and thus one ligated adapter. (B) rAAV2-GFP genomes were extracted using the QIAamp MinElute Virus Spin Kit and the ssDNA was heated to 95°C. The buffer for the double-strand synthesis and $MgSO_4$ were added either before (left) or after the heat shock (right). Following double-strand synthesis, library preparation, and SMRT sequencing, the read lengths and DNA states were analyzed. In addition to the expected full-length fill-ups at approximately 6.7 kb, a lot of full-length duplexes, i.e., annealed DNA with a length of approximately 3.3 kb, are visible if the buffer and MgSO4 are added before the heating step. In contrast, almost no duplex DNA is observed if these components are added after the heat shock.

step. For rAAV2-GFP, we tested ssDNA extracted via both the Qiagen kit and protease treatment. For rAAV9-mGL, only protease-digested samples were included. The percentages of obtained full-length genomes are summarized in Table 2, and the read length plots for the samples extracted with the protease are shown in Figure 4. A consistent trend was observed across all samples: the standard protocol resulted in the lowest percentage of full-length genomes (rAAV2-GFP column based: 37%, rAAV2-GFP protease based: 40%, rAAV9-mGL: 18%), whereas omitting the entire repair and A-tailing step showed slightly better results (rAAV2-GFP column-based: 41%, rAAV2-GFP protease-based: 43%, rAAV9-mGL: 22%). Skipping only the 65°C step resulted in the highest percentage of full-length genomes (rAAV2-GFP column based: 51%, rAAV2-GFP protease based: 51%, rAAV9-mGL: 47%) and showed the best agreement with mass photometry data (Figure S5). The different extraction methods for rAAV2-GFP showed comparable results. For rAAV9-mGL, the difference between the standard protocol and omitting the A-tailing step was most prominent. To ensure that omitting the 65°C step did not compromise contamination detection, we compared the percentages of bases mapping to reference sequences of potential contaminants and observed only minor differences (Table S2).

DISCUSSION

In gene therapy, monitoring the rAAV payload is crucial to ensure the safety and efficacy of the treatment. Due to its high accuracy and long read lengths,[18] SMRT sequencing seems to be a particularly well-suited technique. Nevertheless, library preparation involves various steps that can introduce biases and thus impact the output data. To improve the QC of encapsidated DNA, we investigated the impact of individual library preparation steps and propose an adapted protocol.

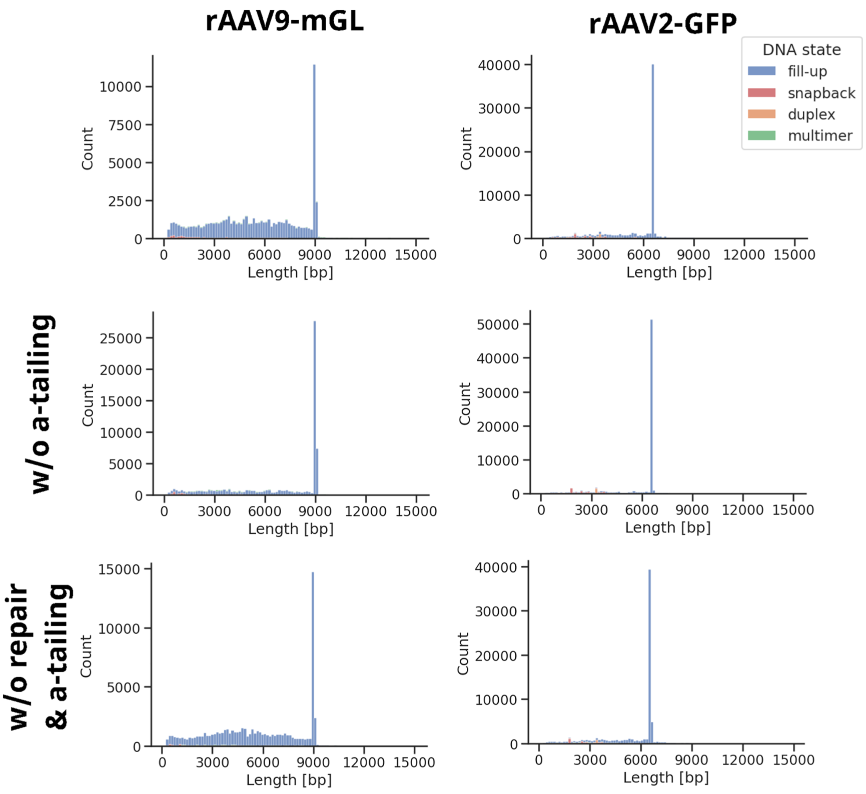

Figure 4. Influence of the repair and A-tailing step on read length distribution

ssDNA from rAAV2-GFP and rAAV9-mGL was extracted using protease treatment. The library preparation was performed according to PacBio's protocol, omitting the 65°C step for A-tailing or without the entire repair and A-tailing procedure. After SMRT sequencing, the read lengths were analyzed. Full-length fill-ups are expected at approximately 6.7 kb and 9.1 kb for rAAV2-GFP and rAAV9-mGL, respectively.

To sequence the DNA, it first must be extracted from the capsids. This step can already have a substantial impact on the genomes. When using the column-based QIAamp MinElute Virus Spin Kit, the measured ssDNA concentration was more than 2.5 times the expected amount for rAAV samples with a titer of $\sim 2 \times 10^{13}$ vg/mL, irrespective of whether the capsid was from AAV9 or AAV2 derived. For rAAV2-GFP with a titer of $\sim 6 \times 10^{12}$ vg/mL, the measured amount was 1.8 times as high as anticipated. In contrast, using only a protease digest to release the ssDNA resulted in the expected amount for all samples (Figure 2A). This difference might be attributed to the following: AAVs can package genomes of both polarities with equal frequency[31] and these strands can anneal, thereby forming dsDNA.[32] On the column of the Qiagen kit, the vector genomes come into close proximity to each other, which facilitates annealing or the formation of other multimeric structures. This could occur more frequently with increasing titer and, consequently, higher genome concentrations. According to the manufacturer of the Qubit ssDNA assay kit, the dye for labeling the nucleic acids cannot distinguish between ssDNA and dsDNA.[33] A spiking experiment revealed that for dsDNA, the ssDNA kit detected approximately 4.5 times the actual input amount (Table 1). Consequently, the obtained rAAV ssDNA amount cannot be determined reliably when using column-based extraction due to an overestimation that increases with the titer. Nevertheless, annealing would also be expected for samples extracted using only protease treatment. Since both extraction methods employ the same digestion protocol, they should release the rAAV ssDNA equally well. While DNA loss may occur during column purification, the protease protocol does not include any purification steps. Therefore, equal or even higher ssDNA amounts might be expected for this approach. However, this was not observed; instead, the measured values corresponded to the expected ssDNA amounts. Consequently, the issue of dsDNA formation that impairs the Qubit ssDNA concentration measurement can be circumvented or at least mitigated by using only a protease treatment for extraction. Interestingly, while rAAV2-GFP ssDNA showed mainly the expected length on the Fragment Analyzer irrespective of the extraction method, the result for rAAV9-mGL exhibited a dependency on the employed extraction protocol (Figure 2B). When using the column-based approach, the main signal was located at approximately 25 kb, and its intensity decreased only slightly with increasing dilution, even with a 1:4 dilution resulting in a lower titer than rAAV2-GFP (Figure S2A). If the protease was used instead of the kit, a signal at 25 kb remained visible but the main peak was located at the expected length. This result is surprising, since formamide, which was used for the length analysis for ssDNA and which is known to denature DNA,[34] was unable to resolve the signal at 25 kb which is most likely caused by multimeric structures of non-covalently linked rAAV genomes. This observation is in line with the difficulties in converting the ssDNA to dsDNA which only worked for the 1:4 diluted rAAV ssDNA sample but was much better for the protease-extracted DNA (Figure S2B). It appears that neither formamide, the heating step prior to double-strand synthesis, nor the strand displacement activity of Bst 3.0 could resolve the assumed multimers. Thus, they are likely not the product of simple annealing but rather the result of other interactions. The magnitude of these interactions seems to correlate with the concentration, as higher dilutions led to a slight decrease in the multimeric signal, and enabled double-strand synthesis (Figure S2). However, as this problem did not occur for rAAV2-GFP, it appears to be additionally dependent on the sequence of the payload. Guanine quadruplexes (G4), which are widespread across the genomes of all organisms and are also known to occur in AAV ITRs, exhibit high thermal stability.[35,36] DNA regions with clusters of 2–5 guanosines separated by 1–7 nucleotides (G(≥2)-N1–7-G(≥2)-N1–7-G(≥2)-N1–7-G(≥2)) can form these

structures.[35] Here, 4 guanines are linked through 8 Hoogsteen hydrogen bonds to form a planar square, stabilized by a central monovalent cation such as K^+, Na^+, or Li^+. The stacking of these so-called G-tetrads results in G4.[34,37,38] These structures, which can be formed intra- or intermolecularly,[39,40] play a pivotal role in regulating biological processes. For instance, they can interfere with elongation during transcription,[35] and thus potentially also with our double-strand synthesis. Analyzing the rAAV2-GFP and rAAV9-mGL genomes for putative G4-forming sequences resulted in 16 hits without overlaps and 743 hits including overlaps for rAAV2-GFP (Figure 1). In contrast, 22 hits without overlaps and 8,269 hits including overlaps were identified for rAAV9-mGL, and they exhibited higher G-scores, indicating a greater likelihood of forming G4 structures compared to the hits for rAAV2-GFP. Therefore, it seems plausible that rAAV9-mGL ssDNA forms G4s and that this behavior is exacerbated by increasing concentrations. Furthermore, sodium ions within the buffer of the QIAamp MinElute Virus Spin Kit might contribute to this. This hypothesis could explain the sequencing results shown in Figure 2C. If rAAV9-mGL DNA is extracted using the column-based method, unresolvable G4s are formed, preventing conversion to dsDNA (Figure S2B) and subsequently to SMRTbell templates. Only short fragments within the sample that do not form such secondary structures can be converted and sequenced. These are either fragments containing ITRs that can be filled up (fill-ups), fragments containing other secondary structures that can serve as primers (snapbacks), or fragments that anneal to each other, are filled up by Bst 3.0, and are trimmed in the repair and A-tailing step (duplexes). In contrast, rAAV9-mGL DNA extracted via protease treatment might not or only at very low levels form G4s and can thus be sequenced. For rAAV2-GFP, which appears less prone to G4 formation, there is no substantial difference in the sequencing results depending on the extraction method. Taken together, we recommend using protease treatment for rAAV ssDNA extraction. First, measurement of the obtained DNA amount is more accurate, enabling a more precise input of DNA in subsequent reactions. Second, this approach could reduce certain biases. As stated in the manufacturer's protocol, fragments smaller than 200 nucleotides are lost with the QIAamp MinElute Virus Spin Kit. Furthermore, we observed a significant influence of the extraction method on the resulting data which might be dependent on the sample concentration and formation of strong secondary structures. Third, the protease digest is approximately four times faster than the column-based approach.

Following the extraction, the rAAV ssDNA has to be converted to dsDNA. Since annealing might introduce biases due to molecules that cannot find an annealing partner or those forming heteroduplexes being trimmed or filled in during the repair step, we decided to take a similar approach to Zhang et al.,[21] namely the synthesis of the second strand using the 3′ ITR as a primer. Since the ITRs are essential for viral packaging, any encapsidated sequence should contain at least an ITR at the 3′ end[6] and thus be converted into dsDNA. However, the introduction of artifacts using this method cannot be entirely excluded and warrants further investigation. In our protocol, Bst 3.0 was used to synthesize the second strand following a heat-shock step to denature annealed genomes. While adding the enzyme buffer and $MgSO_4$ before the heating step resulted in an increase in duplexes and thus annealed rAAV2-GFP genomes, adding them after the heat-shock led to almost exclusively fill-ups (Figure 3B), irrespective of the ssDNA extraction method. For rAAV9-mGL and rAAV2-mGL genomes extracted via protease digest, no annealing was observed, regardless of the time point of buffer addition. These observations may be attributed to differences in the melting temperature (Tm), which is defined as the temperature where half of the dsDNA is denatured, i.e., dissociated into single strands.[41] The rAAV2-GFP payload has a length of 3,342 bases and a Tm of 91.5°C. Although the rAAV2/rAAV9-mGL DNA is longer (4,565 bases), it has a lower Tm of 89.4°C due to its lower GC content compared to rAAV2-GFP. Evaluation of the influence of the buffer components on the melting temperature using a control DNA resulted in a Tm increase from 91°C to 94.5°C. With 91.5°C, rAAV2-GFP has a slightly higher Tm than the control DNA. Therefore, its Tm in the presence of the buffer components might be around 95°C. In contrast, the melting temperature of rAAV2/rAAV9-mGL is approximately 1.6°C lower than that of the control. It seems likely that for rAAV2-GFP, a considerable portion of the annealed genomes does not melt during the heating step. In contrast, the Tm of rAAV2/rAAV9-mGL is about 2.1°C lower, which might be enough for complete denaturation at 95°C. To avoid introducing bias through unintentional annealing, it is advisable to perform the heat shock without any buffer components. Since the obtained amount of ssDNA in the protease-based extraction was the same regardless of whether buffer was used, we would recommend performing this assay without the addition of buffer and adding the components for dsDNA synthesis after the heat-shock step. However, this is only possible as the DNA sample still contains a proportion of formulation buffer from the protease-based extraction. Heating up ssDNA to 95°C in nuclease-free water (NFW) alone would lead to severe DNA damage.

The next enzymatic reaction in PacBio's library preparation protocol, the repair and A-tailing step, is designed to repair DNA damages, such as abasic sites, nicks, and blocked 3′ ends. Additionally, 5′ overhangs are filled in, 3′ overhangs are removed, and 5′ OH-groups are phosphorylated prior to the generation of single A-overhangs.[19] Our protocol involves double-strand synthesis using Bst 3.0 polymerase, which already results in a 3′A-overhang.[42] Evaluating the necessity of the repair and A-tailing step revealed a substantial influence of these reactions on the output data (Table 2, Figure 4). Irrespective of the extraction method and rAAV sample, the percentage of full-length reads was highest when the 65°C step (i.e., the A-tailing) was omitted and showed the best agreement with mass photometry data (Figure S5). Skipping the entire repair and A-tailing step resulted in only slightly improved values compared to the standard protocol (Table 2). Consequently, it appears that the high temperature of 65°C could be problematic. Zhang et al. demonstrated that elevated temperatures during the A-tailing step in Illumina library preparation

contribute to a bias against AT-rich regions.[43] They hypothesized that this is due to nuclease and polymerase activity during thermal breathing, which is the transient breakage of hydrogen bonds below the Tm. Two enzymes might be relevant in this context. The first is T4 DNA polymerase, which is used for end repair and blunting and exhibits a strong 3′→5′ exonuclease activity. The second enzyme, Taq DNA polymerase, is characterized by a 5′→3′ exonuclease activity and the addition of a single A-overhang. Thermal breathing primarily affects base pairs at the dsDNA ends, particularly A-T pairs.[44] Therefore, AT-rich DNA might be more prone to degradation by the 3′→5′ exonuclease activity of T4 DNA polymerase. Moreover, Taq DNA polymerase could produce unwanted cleavage products and, due to inter- or intramolecular annealing of open ends, also primer extension products.[43,45] Regarding SMRT sequencing, the composition of the DNA and end repair mixes is not published. However, PacBio states that the PreCR repair Mix of New England Biolabs (NEB) was formerly used. The only polymerase included in this mix is Bst full length,[46] which, similar to Taq polymerase, exhibits a 5′→3′ exonuclease activity and adds of a single A-overhang. While this enzyme could fill in gaps and perform A-tailing, it is not able to fill in 5′ overhangs or remove 3′ overhangs during end polishing. In a document from 2014, PacBio stated that T4 DNA polymerase is utilized for this purpose.[47] Thus, it is possible that both enzymes, Bst and T4, are present in the current repair mixes, and consequently, the same issues as reported by Zhang et al. might occur. Although ITRs are generally known for their high GC content,[48] the AAV2 ITRs end with an AA motif and thus after the fill-up using Bst 3.0 with AAA/TTA, that could potentially lead to thermal breathing, which would make them prone to exonuclease attacks, unwanted cleavage or primer extension products. Moreover, when present in dsDNA, such as the product of our dsDNA synthesis, the ITRs can adopt two possible conformations: either annealing with the complementary strand, resulting in blunt ends, or forming an intramolecular structure, leading to a molecule with two T-shaped hairpins at one end. It is also likely that the conformation interconverts between these two states. Furthermore, ITRs are known for their instability during plasmid production in bacteria, which might partly be attributed to nuclease attacks.[49] Thus, it seems conceivable that, at elevated temperatures during A-tailing, the ITRs start to "breath" (i.e., undergo conformational changes), leading to the issues described previously for AT-rich sequences. Following this hypothesis, omitting the 65°C step—and thus the A-tailing—should avoid the formation of artificial fragments, which aligns with the higher percentage of full-length genomes detected for these samples (Table 2). However, skipping the entire repair and A-tailing step only yields slightly more reads at the expected length than following the standard protocol. A possible explanation for this observation might be that, after dsDNA synthesis, a proportion of the full-length molecules is not in a state accessible for adapter ligation. These could comprise genomes that were not completely filled up by Bst 3.0 or those lacking a phosphorylated 5′ OH group. Without the repair step, these molecules would be lost during the nuclease treatment, leading to an overrepresentation of fragments in the final data. This overrepresentation might be further exacerbated by the bias against larger fragments on the sequencer.[50,51] Another explanation could be the occurrence of a low frequency of abasic (AP) sites in the rAAV ssDNA. Since DNA polymerases normally stall when encountering such sites,[52] they might impair double-strand synthesis by Bst 3.0, leading to incomplete fill-ups. During the repair step, the DNA backbone at AP sites gets nicked. If there is no A-tailing, no SMRTbell adapters can be ligated to the resulting fragments as they lack an A-overhang. Conversely, with the A-tailing step, they would be amenable to SMRTbell template generation. Concerning the detection of contaminants, only minor differences were observed between performing and omitting the repair and/or A-tailing. Taken together, when sequencing rAAVs and performing dsDNA synthesis with a polymerase capable of adding an A-tail, it appears advantageous to include the repair step, but to skip the incubation at 65°C.

In conclusion, we show that the different steps during rAAV library preparation have a substantial impact on the final data. After a careful evaluation of the influence of the ssDNA extraction method, the conversion to dsDNA, and the repair and A-tailing step, the following adaptations to the standard protocol are recommended: perform a protease digest without the addition of any buffer for the extraction of the rAAV ssDNA from the capsid. For conversion to dsDNA, double-strand synthesis via Bst 3.0 is recommended in order to avoid biases introduced by annealing. The necessary reagents for the polymerase should be added following the heat shock. Concerning the repair and A-tailing step, it is suggested to skip the latter one, i.e., the incubation at 65°C, if a polymerase like Bst 3.0 that adds a 3′ A-overhang has been used. This adapted protocol helps to improve the generation of unbiased sequencing data and thus contributes to enhanced QC of rAAV-based gene therapies.

MATERIALS AND METHODS

rAAV constructs

rAAV2-GFP comprises an AAV2 capsid containing a payload of 3,342 bases encoding EGFP. It is a commercially available vector produced by Sirion Biotech GmbH (Gräfelfing, Germany) via triple transfection in HEK293T cells. After cell lysis with Triton and PEG precipitation, vectors were purified via chromatography using CaptureSelect AAVX resin and iodixanol gradient centrifugation. The final formulation buffer contained 0.001% Poloxamer 188 in PBS. rAAV9-mGL and rAAV2-mGL contain a 4,565-base long genome encoding mGL inside an AAV9 or AAV2-derived capsid, respectively. They were produced in-house by triple transfection in HEK293T cells. Cells were lysed using Triton, and the crude lysate was treated with Benzonase. For purification, the rAAVs were captured on CaptureSelect AAVX (for rAAV2-GFP) or AAV9 (for rAAV9-mGL) resin and polished with anion-exchange chromatography with POROS XQ in both cases. The final formulation buffer consisted of 0.001% Poloxamer 188 in PBS for both rAAV samples.

DNase I treatment

DNase I digestion was performed according to the protocol "Preparing multiplexed AAV SMRTbell libraries using SMRTbell prep kit 3.0" (102-126-400) provided by PacBio.

ssDNA extraction

To extract ssDNA from the vectors, either the QIAamp MinElute Virus Spin Kit (QIAGEN, Hilden, Germany) or a protease-based method was used. For the former, 100 μL of rAAV sample was filled up with NFW to a final volume of 200 μL. The extraction was performed according to the manufacturer's manual, with the exception that no carrier RNA was added. Finally, the ssDNA was eluted in 50 μL buffer AVE (provided with the kit). For the protease-based method, 12.5 μL of protease from the aforementioned Qiagen kit was added per 100 μL of rAAV sample. The mixture was briefly vortexed and incubated at 56°C for 15 min.

ssDNA concentration measurement

The concentration of the obtained rAAV ssDNA was measured with the Qubit 4 Fluorometer using the Qubit ssDNA assay kit (Thermo Fisher Scientific, Waltham, Massachusetts, US) according to the manufacturer's instructions.

Determination of obtained/expected ssDNA amount

The expected ssDNA amount was calculated based on the vg titers according to Equation 1.

$$expected\ ssDNA\ amount[g] = \frac{amount\ of\ used\ vgs}{6.022e^{23}} * (length\ of\ ssDNA[nt]) * 307.97\frac{\frac{g}{mol}}{bp} + 18.02\frac{g}{mol} \quad \text{(Equation 1)}$$

To determine the ratio of obtained to expected ssDNA amount, the concentration of the extracted ssDNA was quantified with the Qubit ssDNA assay kit, and Equation 2 was applied.

$$\frac{obtained\ ssDNA\ amount}{exp\ ected\ ssDNA\ amount} = \frac{measured\ ssDNA\ concnetration * voiume}{expected\ ssDNA\ concentration} \quad \text{(Equation 2)}$$

Double-strand synthesis

To denature annealed genomes, the ssDNA samples were incubated for 5 min at 95°C and then immediately placed on ice. For the synthesis, 16 units (u) of Bst 3.0 (NEB, Ipswich, Massachusetts, US) per 500 ng ssDNA in 1× Isothermal Amplification Buffer II, 8 mM $MgSO_4$ (in total), and 1.4 mM of each dNTP were adjusted to a final volume of 50 μL with NFW. The mixture was incubated at 65°C for 1 h, followed by 5 min at 80°C. Where indicated, the buffer and $MgSO_4$ were added to the ssDNA prior to the 95°C step.

Library preparation and sequencing

Following double-strand synthesis, the standard protocol "Preparing multiplexed AAV SMRTbell libraries using SMRTbell prep kit 3.0" provided by PacBio was implemented starting from step 5, with the following deviations: the bead ratio for purification was adjusted from 1.3× volume/volume to 2.0×, to minimize the loss of short DNA fragments. Additionally, the incubation times for bead binding and elution were extended to 15 min. For evaluation of the repair and A-tailing, samples were either processed according to the standard protocol, with the 65°C incubation omitted ("without A-tailing"), or by skipping the entire step. In the latter case, instead of adding the DNA and end repair mix, the respective samples were filled up to the volume required for the next step with low TE buffer. The resulting SMRTbell libraries were prepared for sequencing according to the sample setup recommendations from SMRT Link and run on a Sequel IIe instrument (Pacific Biosciences, Menlo Park, US) using the standard settings for the "Adeno-associated virus" application.

Bioinformatic analysis

The output files of SMRT sequencing contain only HiFi reads, i.e., reads with a Phred score of at least 20, and thus, no filtering is necessary. Using pbmm2 (v.1.16.0), a minimap2[53] wrapper optimized for PacBio data, the obtained reads were aligned against the plasmid sequences containing the vector genomes and sequences of possible contaminants. These included human DNA (GenBank: GCA_000001405.15), *E. coli* DNA (GenBank: GCA_904425475.1), and a generic RepCapHelper sequence (retrieved from Addgene: pDGM3B), which is for the serotype 3b. Comparison between the cap genes for AAV3b to AAV2 and AAV9 using the BLAST algorithm revealed more than 80% identity. This is well above the compressed-gap sequence identity of pbmm2, which is 70%. Therefore, this sequence was used as a generic reference for the different rAAV samples in this work.

With pysam[54] (v.0.19.1) the aligned bam files were read in pandas[55] (v.1.4.1) DataFrames. To determine the DNA state, the following assumptions were made: duplexes exhibit a very low percentage of soft-clipping. We set the threshold to <10%. Fill-ups should have about 50% soft-clipping (range: 40%–60%) with the ITR in the symmetry axis and one supplementary alignment. Snapbacks are characterized similarly to fill-ups but lack an ITR in the symmetry axis. In contrast, multimers are defined by having at least 75% soft-clipping and more than three supplementary alignments. To generate the read length plots of the primary reads, seaborn[56] (v.0.11.2) was used. For the full-length analysis, the number of reads beginning within 50 bp of the expected start position, ending within 50 bp of the expected stop position and having twice the length ±150 bp of the full-length rAAV genome was divided by the total number of rAAV reads.

Length determination

The lengths of the extracted rAAV ssDNA and the dsDNA after second-strand synthesis were determined using a Fragment Analyzer 5300 instrument (Agilent Technologies, Santa Clara, US). For dsDNA, the DNF-492 Large Fragment Kit was employed according to the manufacturer's protocol. Concerning ssDNA, a custom method was applied which is the subject of a forthcoming publication and is thus not detailed herein.

Melting temperature determination

To evaluate the influence of the buffer used for dsDNA synthesis on the melting behavior of DNA, a plasmid was linearized and purified

using PacBio's magnetic beads to remove buffer components that might affect the melting temperature. Isothermal Amplification Buffer II and $MgSO_4$ were added to reach the identical final concentrations employed for dsDNA synthesis. In the presence of 1× SYBR Green I (Thermo Fisher Scientific, Waltham, Massachusetts, US), the melting curves were determined on a LightCycler 480 Instrument II (Roche Diagnostics GmbH, Mannheim, Germany) applying the standard melting curve protocol for SYBR Green I, omitting the preceding amplification cycles.

Identification of putative quadruplex-forming sequences

For the identification of putative quadruplex-forming sequences in the rAAV2-GFP and rAAV2/rAAV9-mGL genomes, the QGRS Mapper tool[22] was used with the following parameters: maximum length: 30, min. G-group: 2, loop size: 0–36.

Mass photometry

Mass photometry measurements were performed as described by Ebberink et al.[57] with the following modifications: prior to measurement, rAAV stock solutions were pre-diluted in PBS (Gibco) to a final concentration of 5×10^{11} capsid proteins/mL. Measurements were performed using single-use sample carriers (Refeyn Ltd). Data acquisition was carried out on a SamuxMP Auto (Refeyn Ltd.) utilizing the instrument's buffer-free focus finding routine. Movies were recorded for 60 s. Contrast-to-mass conversion was performed using an in-house rAAV preparation of known masses for empty and full capsids.

The boundaries obtained for the empty, partial, and full fractions were transferred to the NGS data using Equation 3. The result was multiplied by two to account for the double-strand synthesis performed during the library preparation workflow, which doubled the original DNA length.

$$ssDNA[bp] = \frac{mass\ at\ border[kDa] - mass\ of\ empty\ capsid[kDa]}{0.33\left[\frac{kDa}{nucletide}\right]}$$

(Equation 3)

The empty fraction in the mass photometry data cannot be entirely captured in the NGS data, as completely empty capsids or very short fragments cannot be sequenced. Therefore, data within the empty fraction boundaries were excluded for both methods, and the sum of the partial and full populations was normalized to 100%.

DATA AND CODE AVAILABILITY

PacBio sequencing data have been deposited to the Sequence Read Archive (SRA) under the accession number PRJNA1347969.

ACKNOWLEDGMENTS

We thank the colleagues in the laboratories at Roche Diagnostics GmbH in Penzberg, Germany for valuable discussions and continuous support. The graphical abstract was created with biorender.com.

AUTHOR CONTRIBUTIONS

Conceptualization, J.M. and J.P.; data curation, J.M.; formal analysis, J.M.; investigation, J.M.; methodology, J.M.; project administration, J.P. and R.R.; resources, M.H. and R.R.; software, J.M. and J.P.; supervision, J.P. and J.H.; validation, J.M.; visualization, J.M.; writing – original draft, J.M.; writing – review and editing, all authors.

DECLARATION OF INTERESTS

J.M., R.R., M.H., and J.P. are employed by Roche Diagnostics GmbH.

SUPPLEMENTAL INFORMATION

Supplemental information can be found online at https://doi.org/10.1016/j.omta.2026.201728.

REFERENCES

1. Wang, J.-H., Gessler, D.J., Zhan, W., Gallagher, T.L., and Gao, G. (2024). Adeno-associated virus as a delivery vector for gene therapy of human diseases. Signal Transduct. Target. Ther. *9*, 78. https://doi.org/10.1038/s41392-024-01780-w.
2. Li, C., and Samulski, R.J. (2020). Engineering adeno-associated virus vectors for gene therapy. Nat. Rev. Genet. *21*, 255–272. https://doi.org/10.1038/s41576-019-0205-4.
3. Yin, L., He, H., Zhang, H., Shang, Y., Fu, C., Wu, S., and Jin, T. (2025). Revolution of AAV in Drug Discovery: From Delivery System to Clinical Application. J. Med. Virol. *97*, e70447. https://doi.org/10.1002/jmv.70447.
4. Duan, D. (2023). Lethal immunotoxicity in high-dose systemic AAV therapy. Mol. Ther. *31*, 3123–3126. https://doi.org/10.1016/j.ymthe.2023.10.015.
5. Beaney, A. (2025). Patient dies in Rocket's Phase II Danon disease gene therapy trial. Clinical Trials Arena. https://www.clinicaltrialsarena.com/news/patient-dies-rocket-danon-disease-gene-therapy-trial/.
6. Wilmott, P., Lisowski, L., Alexander, I.E., and Logan, G.J. (2019). A User's Guide to the Inverted Terminal Repeats of Adeno-Associated Virus. Hum. Gene Ther. Methods *30*, 206–213. https://doi.org/10.1089/hgtb.2019.276.
7. Brimble, M.A., Winston, S.M., and Davidoff, A.M. (2023). Stowaways in the cargo: Contaminating nucleic acids in rAAV preparations for gene therapy. Mol. Ther. *31*, 2826–2838. https://doi.org/10.1016/j.ymthe.2023.07.025.
8. Wright, J.F., and Zelenaia, O. (2011). Vector Characterization Methods for Quality Control Testing of Recombinant Adeno-Associated Viruses. In Viral Vectors for Gene Therapy: Methods and Protocols Methods in Molecular Biology, O.-W. Merten and M. Al-Rubeai, eds. (Humana Press), pp. 247–278. https://doi.org/10.1007/978-1-61779-095-9_11.
9. Analytical approaches to characterize AAV vector production & purification: Advances and challenges BioInsights. https://insights.bio/cell-and-gene-therapy-insights/journal/article/353/Analytical-approaches-to-characterize-AAV-vector-production-purification-Advances-and-challenges.
10. Strong, M.J., Blanchard, E., Lin, Z., Morris, C.A., Baddoo, M., Taylor, C.M., Ware, M.L., and Flemington, E.K. (2016). A comprehensive next generation sequencing-based virome assessment in brain tissue suggests no major virus - tumor association. Acta Neuropathol. Commun. *4*, 71. https://doi.org/10.1186/s40478-016-0338-z.
11. Goodwin, S., McPherson, J.D., and McCombie, W.R. (2016). Coming of age: ten years of next-generation sequencing technologies. Nat. Rev. Genet. *17*, 333–351. https://doi.org/10.1038/nrg.2016.49.
12. Balakrishnan, B., and Jayandharan, G.R. (2014). Basic Biology of Adeno-Associated Virus (AAV) Vectors Used in Gene Therapy. Curr. Gene Ther. *14*, 86–100.
13. Athanasopoulou, K., Boti, M.A., Adamopoulos, P.G., Skourou, P.C., and Scorilas, A. (2021). Third-Generation Sequencing: The Spearhead towards the Radical Transformation of Modern Genomics. Life *12*, 30. https://doi.org/10.3390/life12010030.
14. Wenger, A.M., Peluso, P., Rowell, W.J., Chang, P.-C., Hall, R.J., Concepcion, G.T., Ebler, J., Fungtammasan, A., Kolesnikov, A., Olson, N.D., et al. (2019). Accurate circular consensus long-read sequencing improves variant detection and assembly of a human genome. Nat. Biotechnol. *37*, 1155–1162. https://doi.org/10.1038/s41587-019-0217-9.

15. Rhoads, A., and Au, K.F. (2015). PacBio Sequencing and Its Applications. Genomics Proteomics Bioinformatics *13*, 278–289. https://doi.org/10.1016/j.gpb.2015.08.002.

16. Slatko, B.E., Gardner, A.F., and Ausubel, F.M. (2018). Overview of Next-Generation Sequencing Technologies. Curr. Protoc. Mol. Biol. *122*, e59. https://doi.org/10.1002/cpmb.59.

17. Buermans, H.P.J., and den Dunnen, J.T. (2014). Next generation sequencing technology: Advances and applications. Biochim. Biophys. Acta *1842*, 1932–1941. https://doi.org/10.1016/j.bbadis.2014.06.015.

18. Wang, B. (王博), Ye, K. (叶凯), Jia, P. (贾鹏), Gao, S. (高胜寒), Zhao, H. (赵焕焕), Zheng, G. (郑高洋), and Xu, L. (许林峰) (2025). Long and Accurate: How HiFi Sequencing is Transforming Genomics. Genomics Proteomics Bioinformatics *23*, qzaf003. https://doi.org/10.1093/gpbjnl/qzaf003.

19. Pacific Biosciences (2025). 103-649-300 SMRTbell Library Preparation Overview - Vega System v1.1.

20. https://www.pacb.com/wp-content/uploads/Procedure-checklist-Preparing-multiplexed-AAV-SMRTbell-libraries-using-SMRTbell-prep-kit-3.0.pdf.

21. Zhang, J., Yu, X., Chrzanowski, M., Tian, J., Pouchnik, D., Guo, P., Herzog, R.W., and Xiao, W. (2024). Thorough molecular configuration analysis of noncanonical AAV genomes in AAV vector preparations. Mol. Ther., Methods Clin. Dev. *32*, 101215. https://doi.org/10.1016/j.omtm.2024.101215.

22. Kikin, O., D'Antonio, L., and Bagga, P.S. (2006). QGRS Mapper: a web-based server for predicting G-quadruplexes in nucleotide sequences. Nucleic Acids Res. *34*, W676–W682. https://doi.org/10.1093/nar/gkl253.

23. Bennett, A., Patel, S., Mietzsch, M., Jose, A., Lins-Austin, B., Yu, J.C., Bothner, B., McKenna, R., and Agbandje-McKenna, M. (2017). Thermal Stability as a Determinant of AAV Serotype Identity. Mol. Ther., Methods Clin. Dev. *6*, 171–182. https://doi.org/10.1016/j.omtm.2017.07.003.

24. Tran, N.T., Heiner, C., Weber, K., Weiand, M., Wilmot, D., Xie, J., Wang, D., Brown, A., Manokaran, S., Su, Q., et al. (2020). AAV-Genome Population Sequencing of Vectors Packaging CRISPR Components Reveals Design-Influenced Heterogeneity. Mol. Ther., Methods Clin. Dev. *18*, 639–651. https://doi.org/10.1016/j.omtm.2020.07.007.

25. Silva, R.C.d., de Lima, S.C., dos Santos Reis, W.P.M., de Magalhães, J.J.F., Magalhães, R.N.d.O., Rathi, B., Kohl, A., Bezerra, M.A.C., and Pena, L. (2023). Comparison of DNA extraction methods for COVID-19 host genetics studies. PLoS One *18*, e0287551. https://doi.org/10.1371/journal.pone.0287551.

26. Hawkins, T. (1998). DNA Purification and Isolation Using Magnetic Particles. https://patents.google.com/patent/US5705628A/en.

27. Zhang, J., Guo, P., Yu, X., Frabutt, D.A., Lam, A.K., Mulcrone, P.L., Chrzanowski, M., Firrman, J., Pouchnik, D., Sang, N., et al. (2022). Subgenomic particles in rAAV vectors result from DNA lesion/break and non-homologous end joining of vector genomes. Mol. Ther. Nucleic Acids *29*, 852–861. https://doi.org/10.1016/j.omtn.2022.08.027.

28. King, J.A., Dubielzig, R., Grimm, D., and Kleinschmidt, J.A. (2001). DNA helicase-mediated packaging of adeno-associated virus type 2 genomes into preformed capsids. EMBO J *20*, 3282–3291. https://doi.org/10.1093/emboj/20.12.3282.

29. Maurer, A.C., and Weitzman, M.D. (2020). Adeno-Associated Virus Genome Interactions Important for Vector Production and Transduction. Hum. Gene Ther. *31*, 499–511. https://doi.org/10.1089/hum.2020.069.

30. Gliwa, K., Hull, J., Kansol, A., Zembruski, V., Lakshmanan, R., Mietzsch, M., Chipman, P., Bennett, A., and McKenna, R. (2025). Biophysical and structural insights into AAV genome ejection. J. Virol. *99*, e00899-24. https://doi.org/10.1128/jvi.00899-24.

31. Srivastava, A., Lusby, E.W., and Berns, K.I. (1983). Nucleotide sequence and organization of the adeno-associated virus 2 genome. J. Virol. *45*, 555–564.

32. (2004). Intracellular Viral Processing, Not Single-Stranded DNA Accumulation, Is Crucial for Recombinant Adeno-Associated Virus Transduction. J Virol *78*., 13678–13686. https://doi.org/10.1128/jvi.78.24.13678-13686.

33. Qubit™ ssDNA Assay Kit - FAQs. https://www.thermofisher.com/order/catalog/product/Q10212/faqs.

34. Blake, R.D., and Delcourt, S.G. (1996). Thermodynamic Effects of Formamide on DNA Stability. Nucleic Acids Res. *24*, 2095–2103. https://doi.org/10.1093/nar/24.11.2095.

35. Pathak, R. (2023). G-Quadruplexes in the Viral Genome: Unlocking Targets for Therapeutic Interventions and Antiviral Strategies. Viruses *15*, 2216. https://doi.org/10.3390/v15112216.

36. Bochman, M.L., Paeschke, K., and Zakian, V.A. (2012). DNA secondary structures: stability and function of G-quadruplex structures. Nat. Rev. Genet. *13*, 770–780. https://doi.org/10.1038/nrg3296.

37. Tariq, N., Xu, C., Wang, J., Kume, T., and Macgregor, R.B. (2023). Enhancement of the thermal stability of G-quadruplex structures by urea. Biophys. Chem. *299*, 107043. https://doi.org/10.1016/j.bpc.2023.107043.

38. Jana, J., and Weisz, K. (2021). Thermodynamic Stability of G-Quadruplexes: Impact of Sequence and Environment. Chembiochem. *22*, 2848–2856. https://doi.org/10.1002/cbic.202100127.

39. Sundquist, W.I., and Klug, A. (1989). Telomeric DNA dimerizes by formation of guanine tetrads between hairpin loops. Nature *342*, 825–829. https://doi.org/10.1038/342825a0.

40. Hazel, P., Huppert, J., Balasubramanian, S., and Neidle, S. (2004). Loop-Length-Dependent Folding of G-Quadruplexes. J. Am. Chem. Soc. *126*, 16405–16415. https://doi.org/10.1021/ja045154j.

41. DNA Denaturation - an overview, ScienceDirect Topics. https://www.sciencedirect.com/topics/biochemistry-genetics-and-molecular-biology/dna-denaturation.

42. DNA Polymerase Selection Chart. NEB. https://www.neb.com/en/tools-and-resources/selection-charts/dna-polymerase-selection-chart.

43. Zhang, A., Li, S., Apone, L., Sun, X., Chen, L., Ettwiller, L.M., Langhorst, B.W., Noren, C.J., and Xu, M.-Q. (2018). Solid-phase enzyme catalysis of DNA end repair and 3′ A-tailing reduces GC-bias in next-generation sequencing of human genomic DNA. Sci. Rep. *8*, 15887. https://doi.org/10.1038/s41598-018-34079-2.

44. Jose, D., Datta, K., Johnson, N.P., and von Hippel, P.H. (2009). Spectroscopic studies of position-specific DNA "breathing" fluctuations at replication forks and primer-template junctions. Proc. Natl. Acad. Sci. USA *106*, 4231–4236. https://doi.org/10.1073/pnas.0900803106.

45. Lyamichev, V., Brow, M.A., Varvel, V.E., and Dahlberg, J.E. (1999). Comparison of the 5′ nuclease activities of Taq DNA polymerase and its isolated nuclease domain. Proc. Natl. Acad. Sci. USA *96*, 6143–6148. https://doi.org/10.1073/pnas.96.11.6143.

46. PreCR® Repair Mix | NEB. https://www.neb.com/en/products/m0309-precr-repair-mix?srsltid=AfmBOopVBlDu2VVwyQ-2jkcrXEWaMz4lz5nvG6hK2LTBK59re4lehL67.

47. https://www.pacb.com/wp-content/uploads/2014/04/TemplatePreparation.pdf.

48. Earley, L.F., Conatser, L.M., Lue, V.M., Dobbins, A.L., Li, C., Hirsch, M.L., and Samulski, R.J. (2020). Adeno-Associated Virus Serotype-Specific Inverted Terminal Repeat Sequence Role in Vector Transgene Expression. Hum. Gene Ther. *31*, 151–162. https://doi.org/10.1089/hum.2019.274.

49. Radukic, M.T., Le, D.T., Krassuski, T., Borchert, P., Leach, D.R.F., and Müller, K.M. (2025). Degradation and stable maintenance of adeno-associated virus inverted terminal repeats in E. coli. Nucleic Acids Res. *53*, gkae1170. https://doi.org/10.1093/nar/gkae1170.

50. Tedersoo, L., Tooming-Klunderud, A., and Anslan, S. (2018). PacBio metabarcoding of Fungi and other eukaryotes: errors, biases and perspectives. New Phytol. *217*, 1370–1385. https://doi.org/10.1111/nph.14776.

51. Zhang, X., Davenport, K.W., Gu, W., Daligault, H.E., Munk, A.C., Tashima, H., Reitenga, K., Green, L.D., and Han, C.S. (2012). Improving genome assemblies by sequencing PCR products with PacBio. Biotechniques *53*, 61–62. https://doi.org/10.2144/0000113891.

52. Cuniasse, P., Fazakerley, G.V., Guschlbauer, W., Kaplan, B.E., and Sowers, L.C. (1990). The abasic site as a challenge to DNA polymerase: A nuclear magnetic

resonance study of G, C and T opposite a model abasic site. J. Mol. Biol. *213*, 303–314. https://doi.org/10.1016/S0022-2836(05)80192-5.

53. Li, H. (2018). Minimap2: pairwise alignment for nucleotide sequences. Bioinformatics *34*, 3094–3100. https://doi.org/10.1093/bioinformatics/bty191.

54. Danecek, P., Bonfield, J.K., Liddle, J., Marshall, J., Ohan, V., Pollard, M.O., Whitwham, A., Keane, T., McCarthy, S.A., Davies, R.M., and Li, H. (2021). Twelve years of SAMtools and BCFtools. GigaScience *10*, giab008. https://doi.org/10.1093/gigascience/giab008.

55. McKinney, W. (2010). Data Structures for Statistical Computing in Python. SciPy *445*, 56–61. https://doi.org/10.25080/Majora-92bf1922-00a.

56. Waskom, M. (2021). seaborn: statistical data visualization. J. Open Source Softw. *6*, 3021. https://doi.org/10.21105/joss.03021.

57. Ebberink, E.H.T.M., Ruisinger, A., Nuebel, M., Thomann, M., and Heck, A.J.R. (2022). Assessing production variability in empty and filled adeno-associated viruses by single molecule mass analyses. Mol. Ther., Methods Clin. Dev. *27*, 491–501. https://doi.org/10.1016/j.omtm.2022.11.003.

OMTA, Volume *34*

Supplemental information

Optimizing single molecule, real-time sequencing for enhanced characterization of adeno-associated viral vector genomes

Julia Manz, Raphael Ruppert, Markus Haindl, Jürgen Hubbuch, and Johannes Pschirer

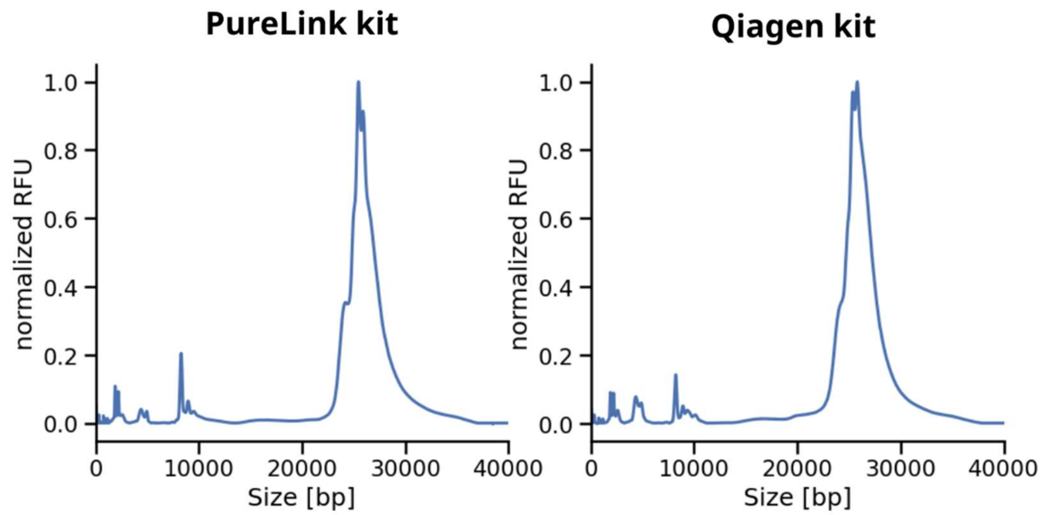

Figure S1: Comparison between the PureLink™ Viral RNA/DNA Mini Kit and the QIAamp MinElute Virus Spin Kit from Qiagen

rAAV2-mGL DNA was extracted using either the PureLink™ Viral RNA/DNA Mini Kit or the QIAamp MinElute Virus Spin Kit. To compare the performance of both kits, the obtained ssDNA was analyzed on a Fragment Analyzer 5300. The RFU values were normalized to the peak with the highest intensity.

A

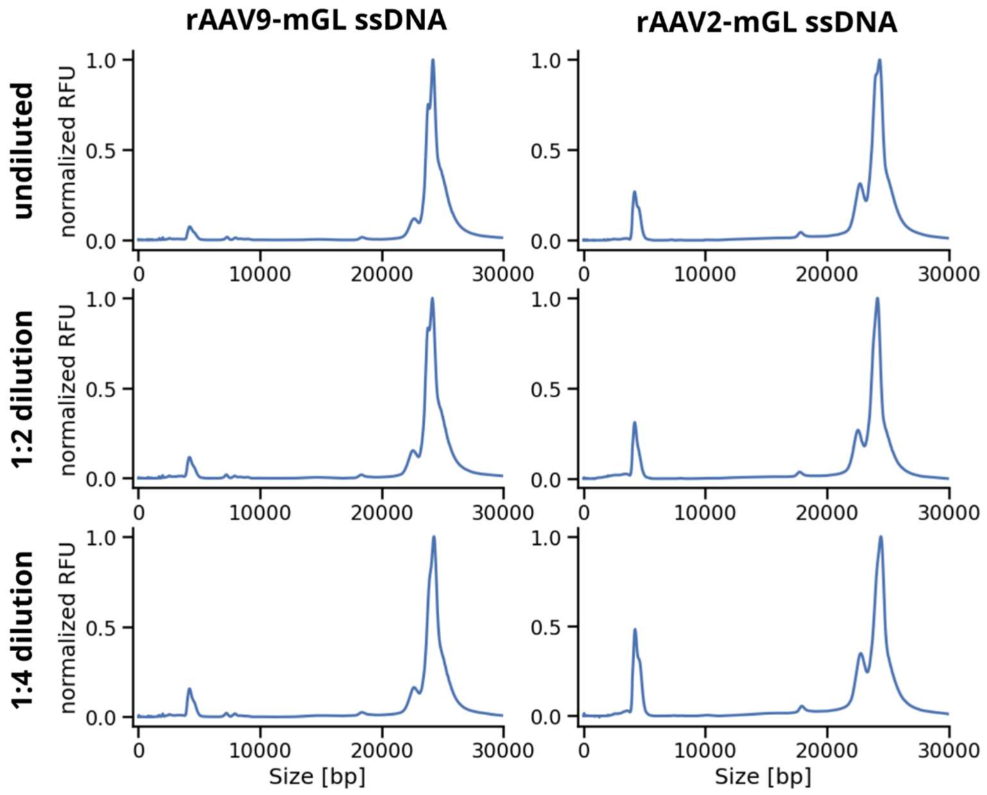
rAAV9-mGL ssDNA
rAAV2-mGL ssDNA
undiluted
1:2 dilution
1:4 dilution
normalized RFU
0.0
0.5
1.0
0
10000
20000
30000
Size [bp]

B

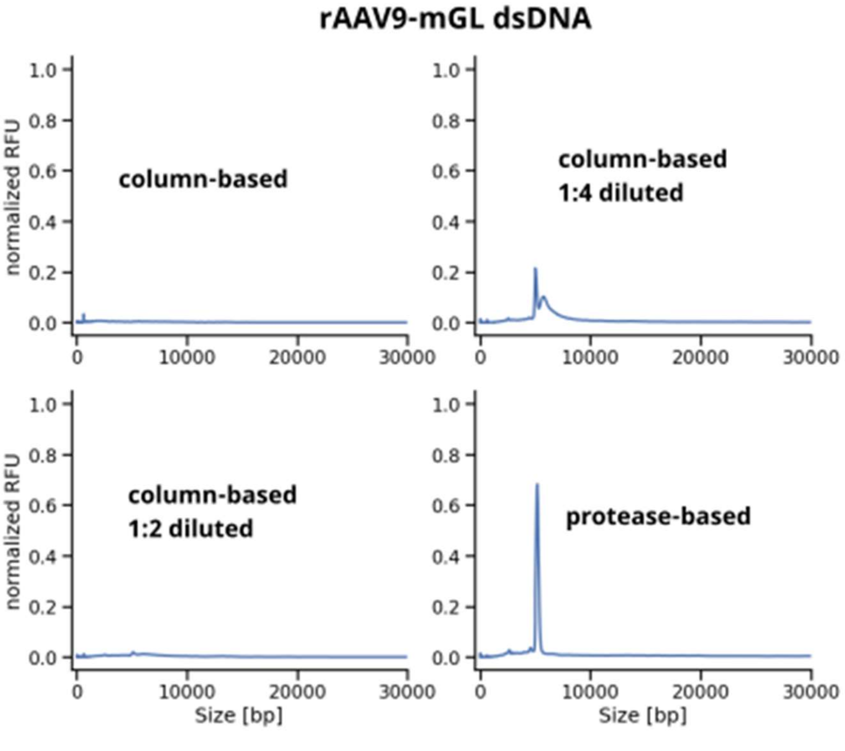
rAAV9-mGL dsDNA
column-based
column-based
1:4 diluted
column-based
1:2 diluted
protease-based
normalized RFU
0.0
0.2
0.4
0.6
0.8
1.0
0
10000
20000
30000
Size [bp]

Figure S2: Investigation of the influence of the titer on the column-based ssDNA extraction

(A) ssDNA from undiluted, 1:2, and 1:4 diluted rAAV9-mGL and rAAV2-mGL samples was extracted using the column-based QIAamp MinElute Virus Spin Kit and analyzed on a Fragment Analyzer 5300. The expected length is approximately 4.5 kb for both vectors. The RFU values were normalized to the peak with the highest intensity. (B) rAAV9-mGL ssDNA was extracted via either protease treatment or the column-based approach. For the latter, different dilutions of the vector were used. The resulting ssDNA served as template for dsDNA synthesis using Bst 3.0. The obtained dsDNA was subsequently analyzed on a Fragment Analyzer 5300.

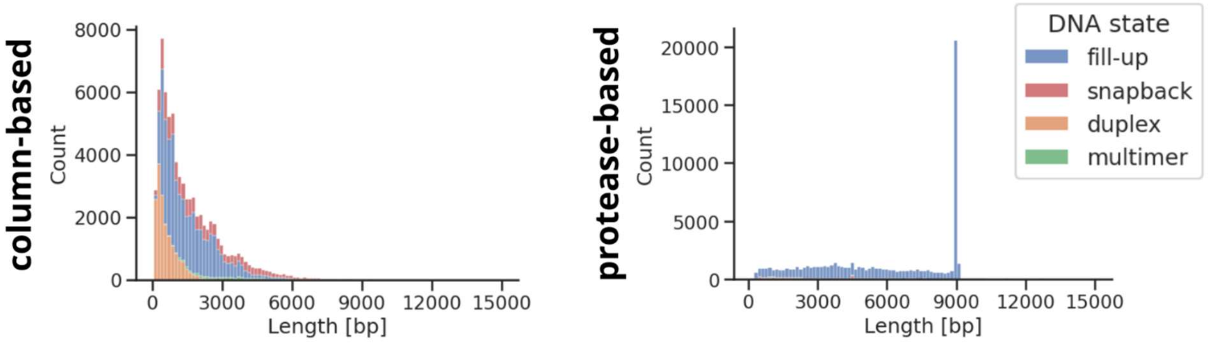

Figure S3: Read length distributions obtained from rAAV2-mGL ssDNA extracted via the column-based or protease-based protocol

rAAV2-mGL ssDNA was extracted using either the column-based QIAamp MinElute Virus Spin Kit or a protease treatment. The plot shows the distribution of the read lengths mapping to the reference plasmid. The DNA states are depicted in different colors. Due to the method used for dsDNA generation, full-length reads are expected to appear as fill-ups with a length of approximately twice the rAAV genome (~9.1 kb).

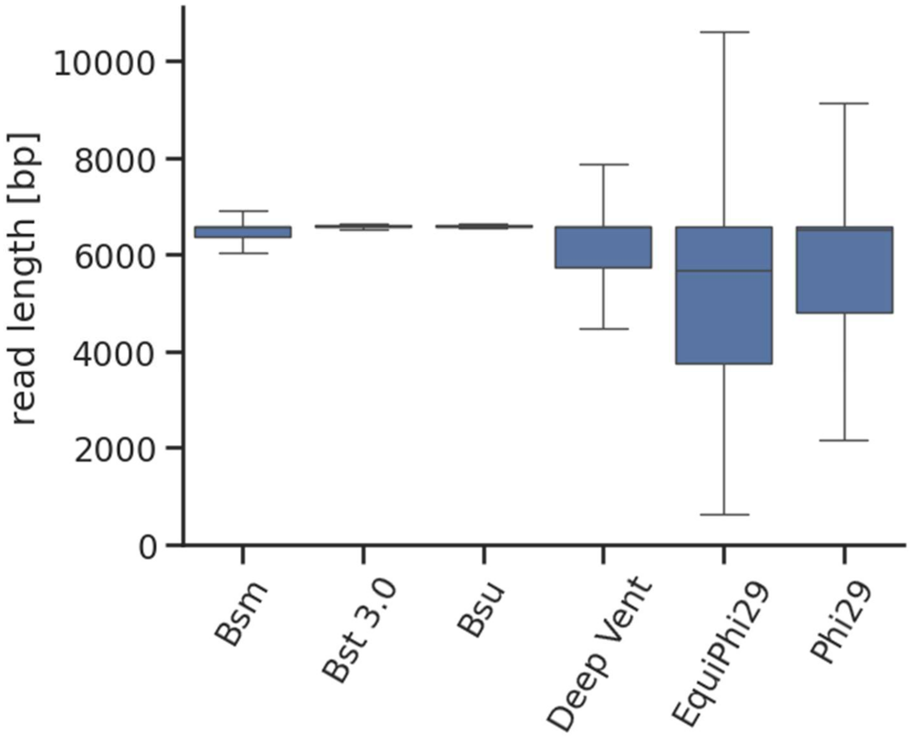

Figure S4: Read length distributions of rAAV2-GFP ssDNA filled up with different polymerases

rAAV2-GFP ssDNA was converted to dsDNA using various polymerases. After library preparation and sequencing, the obtained reads mapping primarily to the reference plasmid were analyzed with regard to their length distribution. The expected read length is ~6.7 kb.

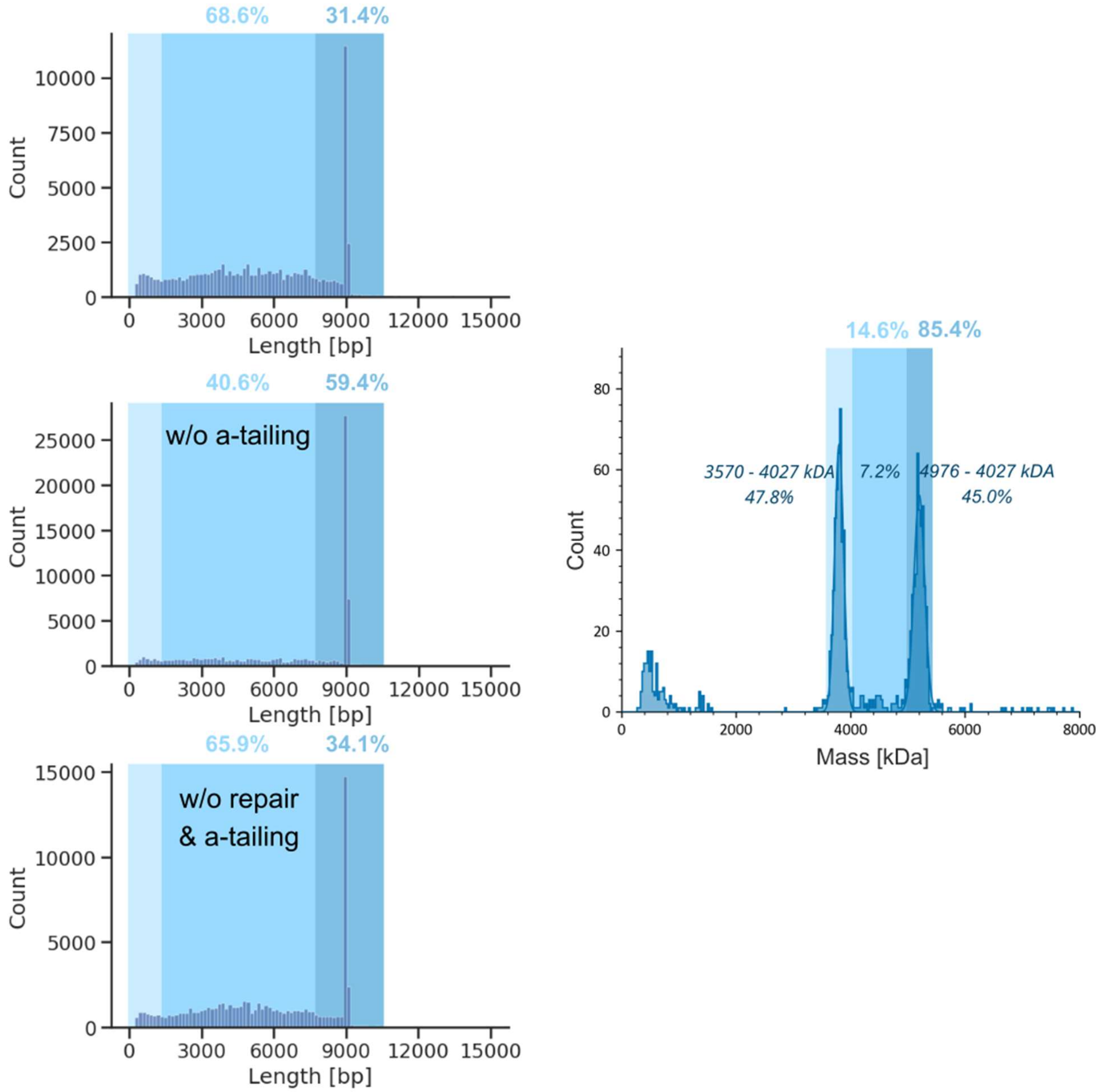

Figure S5: Comparison of NGS data with mass photometry data

rAAV9-mGL ssDNA extracted via protease treatment was prepared for sequencing according to PacBio's protocol, omitting the 65°C step for A-tailing or by skipping the entire repair and A-tailing procedure. After SMRT sequencing, read length distributions were analyzed (left). Additionally, mass photometry was used to quantify the percentage of empty (light blue), partial (mid blue) and full capsids (dark blue) (right). The boundaries obtained via mass photometry were transferred to the NGS data. The percentages of the partial and full fractions excluding the empty one are depicted above each diagram.

Table S1: Percentage of bases fitting to different reference sequences and percentage of full-length rAAV genomes in rAAV2-mGL samples with and without DNase treatment prior to genome extraction

rAAV: recombinant adeno-associated viral vector; pRepCapHelper: generic sequence of contamination with repcap or helper plasmids

		DNase treatment	no DNase treatment
% bases	rAAV	97.5	97.6
	plasmid backbone	1.7	1.6
	pRepCapHepler	0.7	0.7
	human	0.1	0.1
	E. coli	0.0	0.0
	unmapped	0.0	0.0
% full length rAAV genomes		30.0	29.7

Table S2: Percentage of bases fitting to different contamination-related reference sequences

rAAV: recombinant adeno-associated viral vector; pRepCapHelper: generic sequence of contamination with repcap or helper plasmids

rAAV	Repair & A-Tailing	Contamination	% bases
rAAV2-GFP	yes	plasmid backbone	3.1
		pRepCapHelper	0.2
		human	0.2
		E. coli	0.1
		unmapped	0.0

	only repair step	plasmid backbone	1.8
		pRepCapHelper	0.1
		human	0.1
		E. coli	0.1
		unmapped	0.0
	no	plasmid backbone	2.5
		pRepCapHelper	0.2
		human	0.1
		E. coli	0.1
		unmapped	0.0
rAAV9-mGL	yes	plasmid backbone	0.2
		pRepCapHelper	4.6
		human	0.3
		E. coli	0.0
		unmapped	0.0
	only repair step	plasmid backbone	0.2
		pRepCapHelper	3.1
		human	0.1
		E. coli	0.0
		unmapped	0.0
	no	plasmid backbone	0.2
		pRepCapHelper	4.6
		human	0.2
		E. coli	0.0
		unmapped	0.0
